# Himalayan Asphaltum-Driven Synthesis of Gold and Selenium Nanoparticles: Investigation of Antimicrobial Activity and Urolithin Levels

**DOI:** 10.3390/molecules31152649

**Published:** 2026-07-29

**Authors:** Nenad Janković, Nodari Rizun, Srđan Stefanović, Lazar Rakočević, Jovana Matić, Sebastian Baloš, Marijana Kosanić, Nenad Ignjatović

**Affiliations:** 1 Department of Science, Institute for Information Technologies Kragujevac, University of Kragujevac, Liceja Kneževine Srbije 1A, 34000 Kragujevac, Serbia; jovana.todorovic@pmf.kg.ac.rs; 2 PÜRBLACK Inc., 41877 Enterprise Circle N., Temecula, CA 92590, USA; nodari@purblack.com; 3 Institute of Meat Hygiene and Technology, Kaćanskog 13, 11000 Belgrade, Serbia; srdjan.stefanovic@inmes.rs; 4 Department of Atomic Physics, Institute of Nuclear Sciences Vinča, University of Belgrade, Mike Petrovića Alasa 12–14, 11000 Belgrade, Serbia; lazar.rakocevic@vin.bg.ac.rs; 5 Faculty of Technical Sciences, University of Novi Sad, Trg Dositeja Obradovića 6, 21000 Novi Sad, Serbia; sebab@uns.ac.rs; 6 Department of Biology and Ecology, University of Kragujevac, Faculty of Science, Radoja Domanovića 12, 34000 Kragujevac, Serbia; marijana.kosanic@pmf.kg.ac.rs; 7 Institute of Technical Sciences of the Serbian Academy of Science and Arts, Knez Mihailova 35/IV, P.O. Box 377 Belgrade, Serbia; nenad.ignjatovic@itn.sanu.ac.rs

**Keywords:** shilajit, urolithin, nanogold, nanoselenium, antimicrobials

## Abstract

This work examines the utilization of the natural product shilajit resin, sourced from the Himalayas, for the extraction of diverse components suitable to the synthesis of gold nanoparticles (AuNPs) and selenium nanoparticles (SeNPs). The study evaluated the extraction efficiency of **Uro-A**, a polar phenolic component, from shilajit samples utilizing various solvent systems, followed by the eco-friendly synthesis and characterization of AuNPs and SeNPs. The LC-MS/MS analysis demonstrated that the methanol/ethyl acetate (MeOH/EA) extract was particularly effective and contained the highest concentration of **Uro-A**. A robust positive association was discovered between **Uro-A** content in shilajit and the efficiency of nanoparticle production. Sample **2**, with 0.002 ppm **Uro-A**, produced suboptimal yields (15% for AuNPs; 34% for SeNPs), while sample **1**, with 2.504 ppm **Uro-A**, noticeably enhanced the yields to 90% (AuNPs) and 96% (SeNPs). The synthesized AuNPs and SeNPs were characterized using SEM, EDS, PSD, ZP, XRD, FTIR, and XPS techniques, demonstrating spherical particles with an average diameter of around 100 nm. XPS spectra confirmed the elemental form of the obtained AuNPs and SeNPs. Antimicrobial studies revealed that SeNPs exhibit moderate efficacy (MIC = 0.125–1 mg/mL) against bacteria (e.g., *Bacillus subtilis*, *Staphylococcus aureus*) and fungi (e.g., *Penicillium italicum*, *Mucor mucedo*), but higher in comparison to AuNPs (MIC 0.25–5 mg/mL).

## 1. Introduction

Nanotechnology has emerged as a revolutionary domain in contemporary research, providing novel solutions across multiple fields, including health, environmental science, and materials engineering. The capacity to control materials at the nanoscale (1–100 nm) facilitates the creation of innovative materials with distinct physicochemical properties, including elevated surface-to-volume ratios, increased reactivity, and adjustable optical and electrical characteristics [1,2,3]. Metallic and metalloid nanoparticles (NPs), particularly gold (Au) and selenium (Se), have attracted considerable interest for their potential in biomedical applications such as drug administration, imaging, diagnostics, and antimicrobial therapy [4,5].

Gold NPs (AuNPs) are esteemed for their biocompatibility, chemical stability, and simplicity of functionalization, performing them as useful platforms for targeted drug administration and antimicrobial applications. Their surface plasmon resonance characteristics facilitate applications in biosensing and imaging. Selenium NPs (SeNPs) are recognized for their antioxidant capabilities, low toxicity, and intrinsic antibacterial activity, rendering them appropriate for medicinal and nutritional applications [6,7]. The fusion of these NPs with natural bioactive molecules presents a synergistic strategy to extend their efficiency and safety, especially in the fight against microbial diseases. The fabrication of NPs has conventionally depended on physical and chemical techniques, including chemical reduction, laser ablation, and photochemical approaches [8,9,10,11,12]. Nonetheless, these techniques frequently include hazardous chemicals, significant energy expenditure, and ecologically harmful consequences, prompting concerns regarding their sustainability and safety for biological uses. Consequently, green synthesis has emerged as a sustainable, environmentally friendly, and economical alternative to conventional methods, utilizing natural resources—such as plant extracts, microbes, and biomolecules—to produce NPs while reducing environmental impact [13,14]. Green-driven synthesis minimizes the utilization of toxic chemicals while simultaneously improving the biocompatibility of NPs by the integration of bioactive compounds derived from natural sources into their composition [15,16,17,18,19,20,21,22,23]. The long-term crisis of antibiotic resistance, identified by the World Health Organization as a leading global public health threat, has urged extensive research into alternative antimicrobial strategies, with green-synthesized AuNP and SeNPs emerging as prominent candidates due to their effective and diverse antibacterial mechanisms [24,25,26,27].

The incorporation of shilajit-derived biomolecules into the nanoparticle framework may provide additional therapeutic advantages, including improved antibacterial or antioxidant activities, which could extend the intrinsic properties of AuNPs and SeNPs. Shilajit is a resin and natural matter from the Himalayas, created over centuries by the decomposition of plants [28]. AuNPs and SeNPs, synthesized through environmentally sustainable biological methods utilizing plant extracts, microbes, or natural biomolecules, demonstrate superior efficacy against multidrug-resistant (MDR) pathogens in comparison to their chemically synthesized equivalents [29,30]. This enhancement is primarily attributed to the biogenic capping agents—such as polyphenols, flavonoids, and proteins—that confer additional bioactive properties, synergizing with the NPs intrinsic reactivity. Biogenic capping agents during synthesis of NPs prevent aggregation, control size/shape, and enhance stability [31,32]. Bioderived AuNPs exhibit exceptional antibacterial efficacy via various mechanisms, such as the disruption of bacterial cell membranes, inhibition of enzymatic activity, and the production of reactive oxygen species (ROS) that provoke oxidative stress, resulting in DNA damage and protein denaturation in resistant strains such as *Staphylococcus aureus* and *Escherichia coli* [24]. Likewise, SeNPs, typically biosynthesized by bacterial or fungal reductases, are proficient in countering Gram-negative pathogens such as *Pseudomonas aeruginosa* by disrupting biofilm formation and sensing, which are critical factors in chronic infections [26,27]. AuNPs and SeNPs have been the subject of numerous studies due to their satisfactory biocompatibility and suitability for potential application in various fields of medicine [33,34,35]. Despite these advantages, it has been shown that smaller AuNPs can more easily enter cells and accumulate in various organs, including the liver, spleen, and brain, posing potential health risks [36].

This surge in interest stems not only from AMR’s projected toll—estimated at 10 million annual deaths by 2050—but also from the sustainability angle: green methods avoid toxic solvents and reduce environmental footprints, aligning with global calls for One Health approaches [24]. Recent studies highlight synergistic formulations, such as Au-Se nanocomposites or those combined with natural extracts, achieving up to 90% inhibition of MDR biofilms at sub-toxic doses, paving the way for clinical translations in wound dressings, coatings, and therapeutics [26,27].

The primary goal of this study was to demonstrate a fully green and additive-free method for AuNP and SeNP synthesis using shilajit resin as the sole reducing, stabilizing, and capping agent. A method is presented that enables the synthesis of AuNPs and SeNPs with uniform size and unimodal particle size distribution. The resulting shilajit-capped NPs were evaluated against pathogenic bacteria and fungi.

## 2. Results

### 2.1. LC-MS/MS Quantification of the Uro-A and Synthesis of AuNPs and SeNPs Mediated by Shilajit

Table 1 provides measurements of level **Uro-A** for two samples of shilajit (**1** and **2**) and three extracts obtained under three solvent conditions: hexane, EA (ethyl acetate), and MeOH/EA (methanol/ethyl acetate mixture). The values likely represent a quantitative measure of the targeted molecule **Uro-A**.

For sample **1**, this extremely low value of **Uro-A** (0.002 ppm) suggests minimal solubility or extraction of shilajit components in hexane (Figures S2 and S3), a non-polar solvent. **Uro-A** is a polar phenolic compound, which is poorly soluble in non-polar solvents like hexane. Extraction efficacy in EA (Figures S4 and S5) is slightly higher than with hexane, indicating a modest increase in solubility of **Uro-A** (0.01 ppm). In the MeOH/EA solvent system, the content of **Uro-A** is dramatically higher (2.504 ppm; 1252 and 250 times higher than in the hexane and EA extracts, respectively), indicating a high extraction efficiency of the MeOH/EA solvent mixture (Figures S6 and S7). The results highlight MeOH/EA as the most effective solvent for both shilajit samples, with sample 1 showing a significantly stronger response (2.504 vs. 0.209 ppm), suggesting a richer composition in **Uro-A**. High-performance liquid chromatography–mass spectrometry (HPLC-MS) analysis of **Uro-A** and **-B** identified shilajit sample **1** (Table 1) as optimal for synthesizing AuNPs and SeNPs.

### 2.2. Physicochemical Characterization of AuNPs, SeNPs, and Shilajit

**Figure 1:**
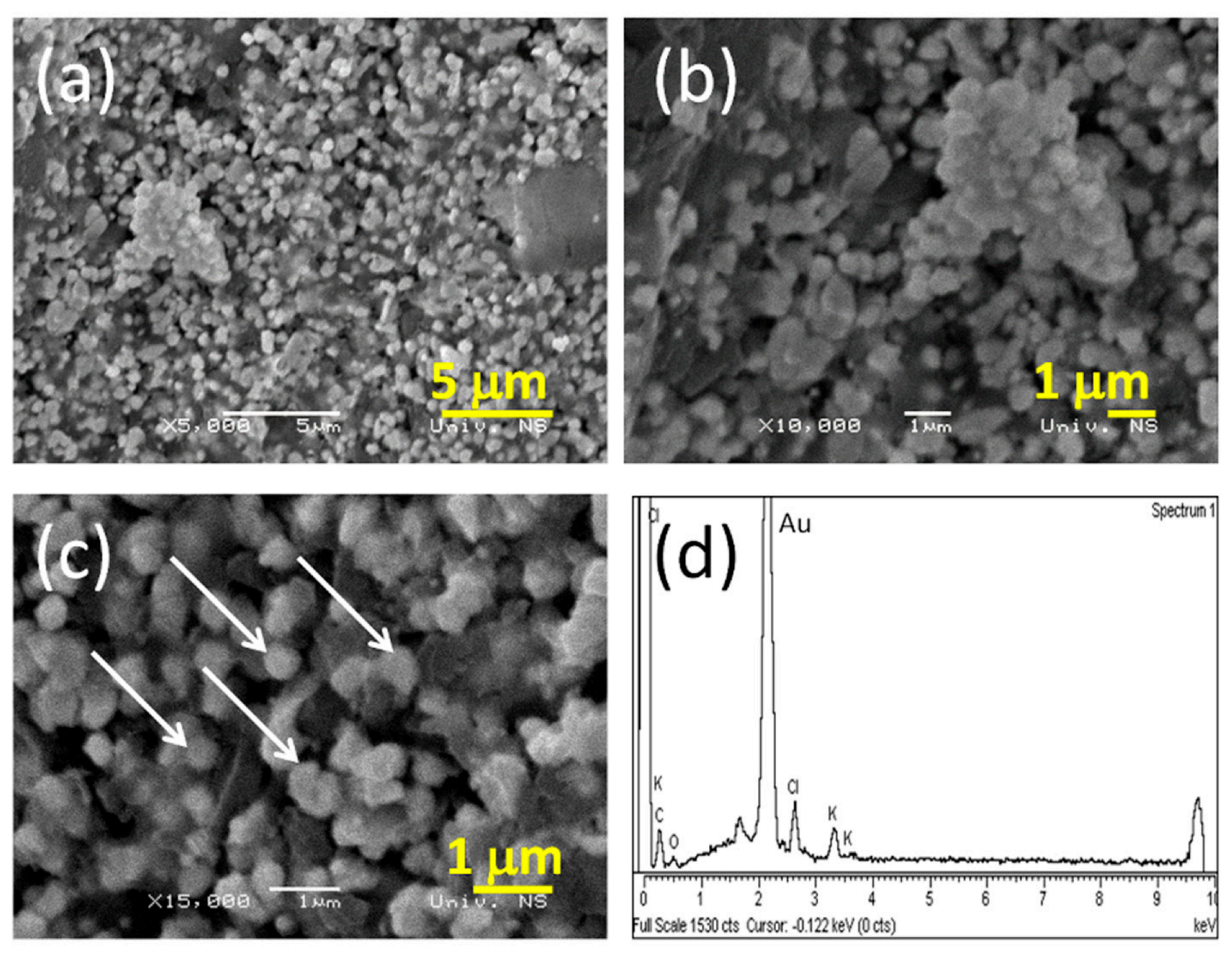
SEM and EDS of obtained gold powder. SEM images were recorded at different magnifications: (**a**) 5000×, (**b**) 10,000×, (**c**) 15,000×, and (**d**) EDS spectra.

The morphology of obtained gold powder is shown in Figure 1a–c at magnifications of 5000×, 10,000×, and 15,000×. The morphology of the obtained gold powder is characterized by relatively uniform particles of sphere-like morphology (Figure 1a). As the magnification increases to 15,000×, the finer details of the surface morphology become more apparent, with at most double and triple agglomerates of individual particles. The particles were in the size range of 80 to 250 nm and are marked with arrows in Figure 1c. A representative EDS spectrum presented in Figure 1d confirms the qualitative nature of the distributed gold particles as well as their presence (Kα at 2.2 keV) (corresponding SEM images are given in Figure 1c). Small amounts of potassium (K) originating from shilajit were detected also [37].

**Figure 2:**
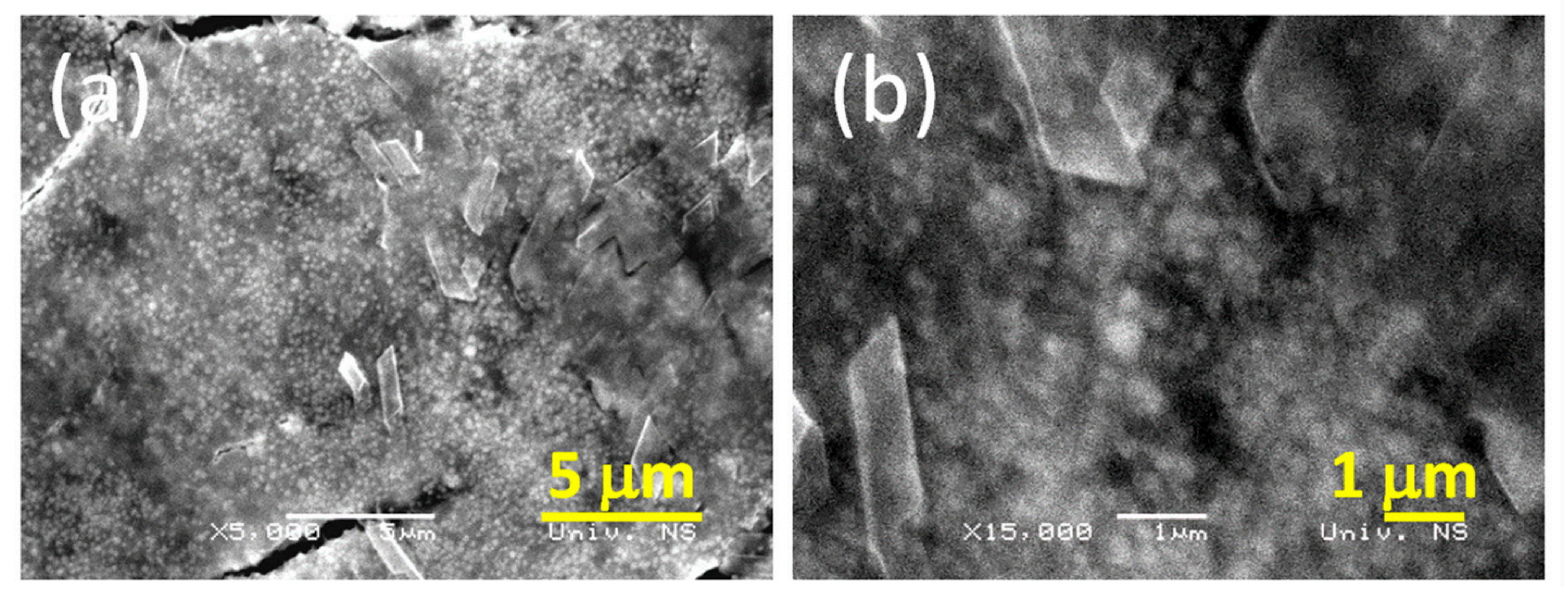

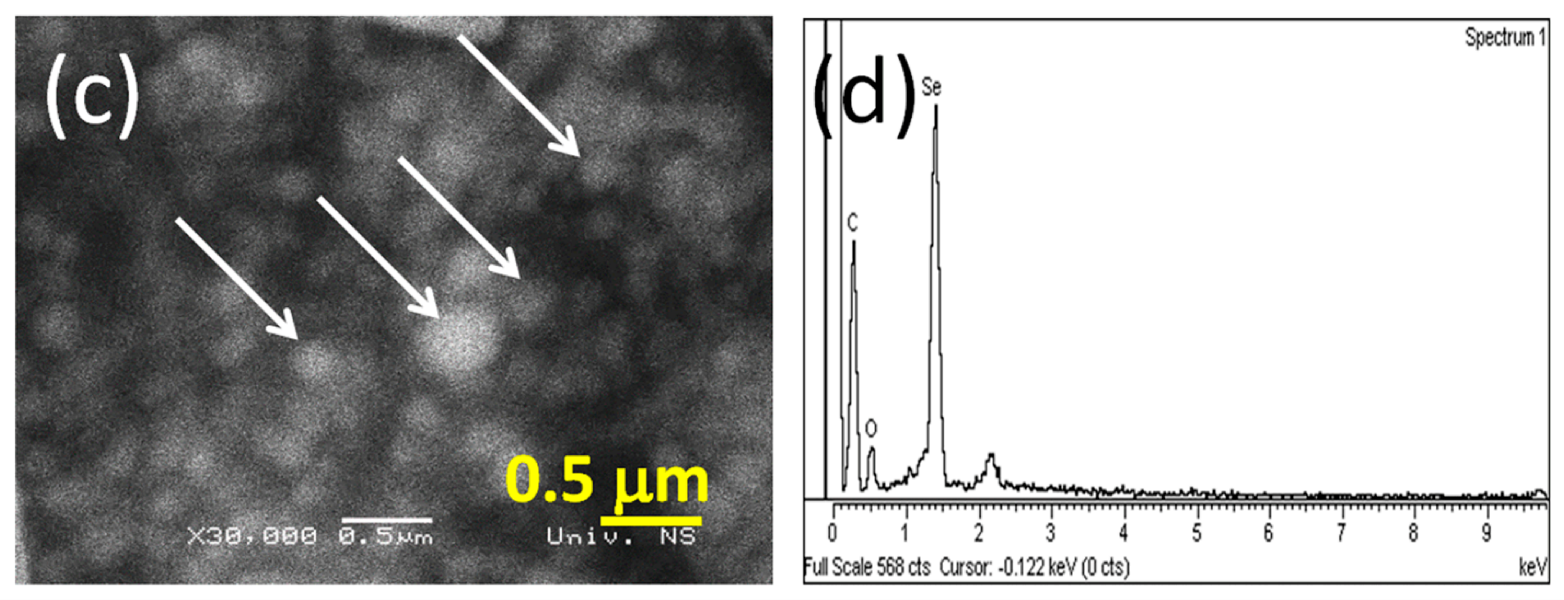
SEM and EDS of obtained selenium powder. SEM images were recorded at different magnifications: (**a**) 5000×, (**b**) 15,000×, and (**c**) 30,000×; (**d**) EDS spectra.

Figure 2a–c show the SEM images of obtained selenium powder at various magnifications. Most powder particles are generally close to spheres. At a magnification of 15,000×, the primary particles are more clearly visible on the powder surface. The SEM image of the surface of the selenium powder exhibited a relatively uniform and spherical-like morphology of the particles (Figure 2b). As the magnification increases to 30,000×, the finer details of the surface morphology become more apparent, with individual particles clearly visible (Figure 2c). The particles were in the size range of 80 to 300 nm (are marked with arrows in Figure 2c). A representative EDS spectrum presented in Figure 2d confirms the qualitative nature of the distributed selenium particles as well as their presence (Kα at 1.3 keV) (corresponding SEM images are given in Figure 2c).

**Figure 3:**
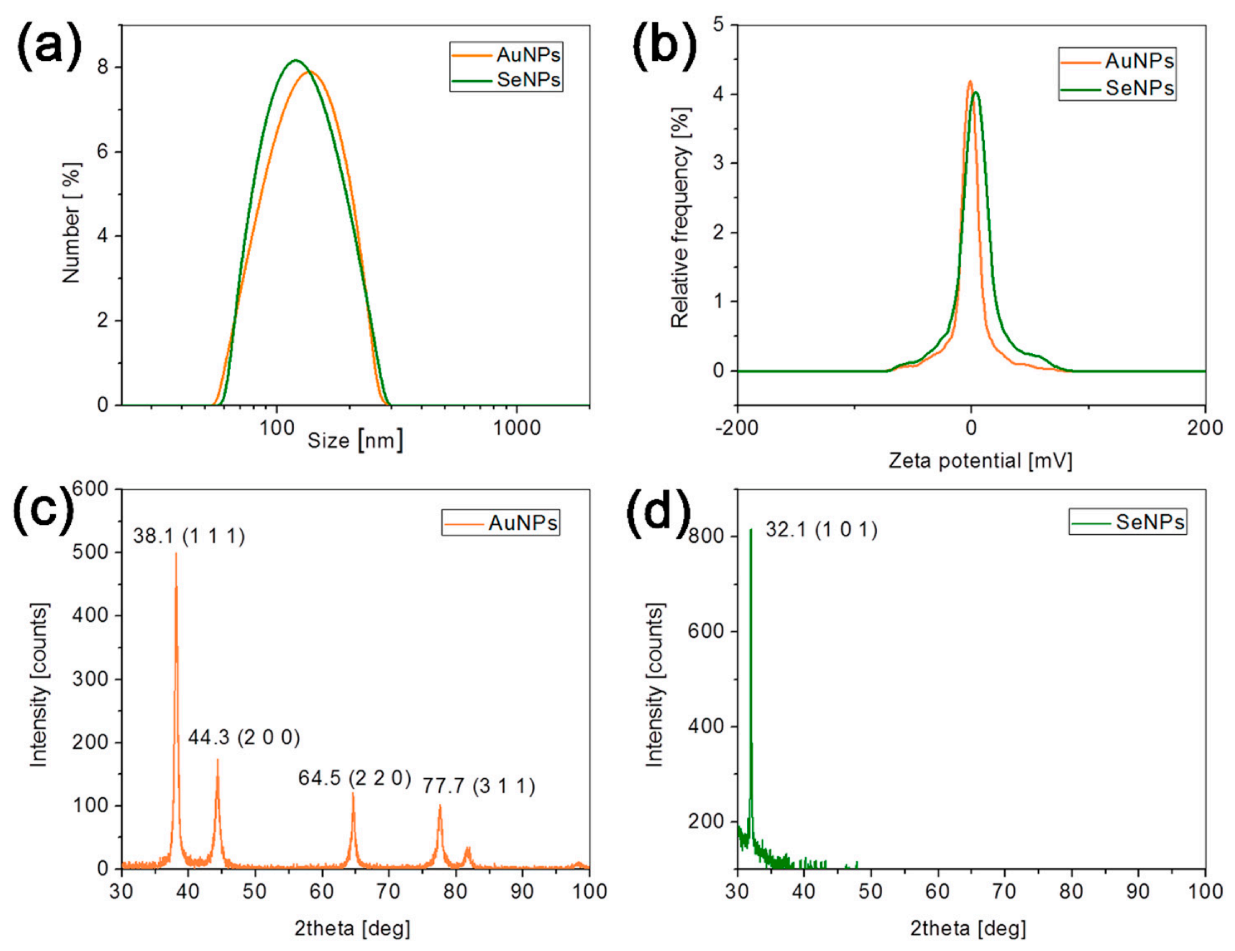
AuNPs and SeNPs: (**a**) Particle size distribution (PSD), (**b**) zeta potential (ZP), (**c**,**d**) X-ray diffraction (XRD). AuNPs: Mean zeta potential −2.2 mV, standard deviation 0.51 mV; SeNPs: Mean zeta potential −4.8 mV, standard deviation 0.82 mV.

The size distribution of the obtained particles (PSD) is shown in Figure 3a and Figure S8. The maximum distribution of both types (AuNPs and SeNPs) of particles was located at about 100 nm. The distribution for each type of particle is unimodal, with a clearly defined single peak (dark orange for Au particles and green for Se particles). The measured zeta potential values were −2.2 mV for AuNPs and −4.8 mV for SeNPs, respectively (Figure 3b). The low zeta potential for NPs between +10 mV and −10 mV indicates a neutral (or near neutral) surface charge and a potential tendency toward agglomeration [38]. Accordingly, agglomeration of primary particles is also confirmed in Figure 1c and Figure 2c. The agglomeration is more noticeable in obtained gold powder than in selenium, most likely due to the slightly lower zeta potential of selenium particles. Absolute zeta potential values below 30 mV may potentially indicate the possibility of agglomeration, which may represent a limitation to successful application [39].

Results of the XRD phase analysis of samples are shown in Figure 3c,d. Crystalline AuNPs are represented by four typical peaks corresponding to the standard Bragg reflections (111), (200), (220), and (311) (Figure 3c). The most intense diffraction peak at 38.1 most likely indicates that the preferred growth orientation of zero-valent gold is fixed in the (111) direction, which is consistent with the results of other authors [40,41]. In accordance with standard database files, JCPDS (file number 893697) as well as the results of other authors, the XRD results revealed that the AuNPs were formed in elemental form by the reduction of the metal ions [42].

The peak you observe at ≈32° 2θ (Cu Kα radiation, λ = 1.5406 Å) is highly characteristic of biogenic, amorphous-to-poorly crystalline selenium NPs and appears repeatedly in the recent literature on plant-, microbe-, or humic-substance-mediated SeNPs (Figure 3d) [43,44]. The characteristic diffractogram shown in Figure 3d indicates the reduction of selenite to elemental selenium NPs (SeNPs), which is in agreement with the results of other authors and with the JCPDS (file no. 83-2437) [45]. The typical limit of detection for XRD is approximately 1–5 wt%. Specific elements from organic molecules, such as C, O or similar, are confirmed in the EDS results (Figure 1d and Figure 2d). However, the XRD results confirmed only the presence of Au and Se phases, so we could approximate that the organic phase content in the AuNP and SeNP samples was less than 5%.

**Figure 4:**
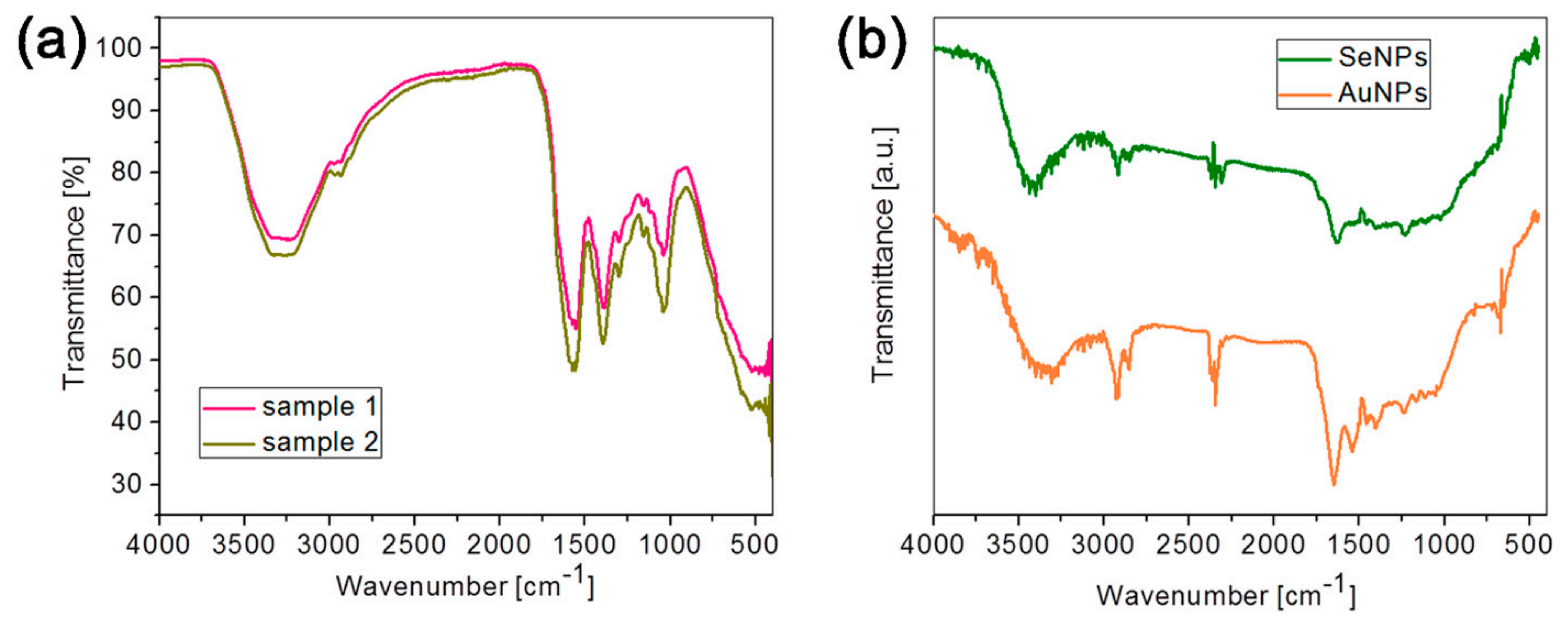
FTIR spectra in range 4000–500 cm^−1^: (**a**) samples 1 (red line) and 2 (green line); (**b**) AuNPs (orange line) and SeNPs (green line).

Overlapped IR spectra of samples of shilajit **1** and **2** (samples **1** and **2**) and AuNPs and SeNPs are presented in Figure 4a,b. Both samples **1** and **2** exhibit similar overall patterns, suggesting they contained common functional groups, consistent with shilajit’s complex composition of organic compounds (e.g., fulvic acid, humic substances, and phenolic compounds). Both samples show a broad absorption band around 3400–3200 cm^−1^, typical of O–H stretching in alcohols, and carboxylic acids or N–H stretching in amides. This is characteristic of shilajit’s polar organic components like fulvic acid and phenolic compounds, which contain numerous -OH and/or -COOH groups [16,46,47]. A band occurs around 1560 cm^−1^, corresponding to C=O stretching in carbonyl groups from humate salt [48]. Multiple overlapping bands are visible, particularly around 1400 cm^−1^ and 1300 cm^−1^, likely due to C–O stretching, O–H bending, or aromatic C–H in-plane bending. These bands reinforce the presence of phenolic and carboxylic acid groups. Specifically, the band at 1393 cm^−1^ presents symmetric stretching of COO^−^ and C–OH stretching of phenolic OH. The C–O stretching band of carbohydrates is found at 1035 cm^−1^ [49].

In Figure 4b, SeNPs (green line) generally show lower transmittance (higher absorption) than AuNPs (orange line) in several regions, suggesting differences in surface composition or stabilization. A broad scattering background was observed in the ATR-FTIR spectra, which is commonly attributed to particle size distribution and variable sample-crystal contact in powder samples, resulting in increased IR scattering and baseline shifts [50]. A broad band is found at both IR spectra at 3400 cm^−1^ that corresponds to an O–H stretch. In both samples is found a band at around 2930 cm^−1^ that corresponds to a C–H stretch [50,51]. The band observed at 2350 cm^−1^ is attributed to symmetric COO^−^ stretching. Bands at 1651 (AuNPs), and 1625 cm^−1^ (SeNPs) indicate conjugated C=O stretching vibration [52,53]. The peaks at 1538 and 1524 cm^−1^ correspond to the spectral region characteristic of N–H bending vibrations [50]. Together with the bands in the range 1600–1650 cm^−1^, 1520–1550 cm^−1^ (C=O stretching and N–H bending of amide groups in proteins, respectively) and at approximately 1250 cm^−1^, these correspond to the amide I, amide II, and amide III bands, respectively [53]. In both samples of NPs, the OH deformation bands can be observed at 1410 cm^−1^, with possible contributions from C–N of primary amides (amide III band) and COO^−^ groups [54]. Based on the literature and consistent with our FTIR results, the capping agents are most likely amide and phenolic compounds [52]. Considering the deeper absorption in SeNPs suggests a richer organic content or stronger binding of capping agents, consistent with selenium’s higher reactivity requiring robust stabilization. This observation corresponds with EDS measurements for carbon content that is higher in SeNPs related to AuNPs (Figure 1d and Figure 2d).

**Figure 5:**
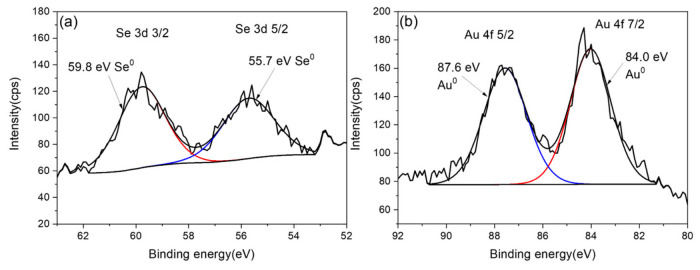
High-resolution XPS spectra of (**a**) Se 3d and (**b**) Au 4f.

Figure 5 presents the high-resolution X-ray photoelectron spectroscopy (XPS) spectra of the Se 3d and Au 4f core levels, which were analyzed to determine the chemical states and electronic environments of selenium and gold in the sample. Figure 5a shows the Se 3d spectrum, which exhibits two distinct peaks at binding energies of 59.8 eV and 55.7 eV. These two peaks correspond to the spin–orbit split components of the Se 3d core level (Se 3d 3/2 and Se 3d 5/2) and are characteristic of elemental, metalloid selenium. The sharpness and positions of these peaks indicate that selenium in the sample exists entirely in its zero-valent, metalloid form, without significant contributions from oxidized or other chemical states [55,56]. Figure 1b shows the Au 4f spectrum, which also shows two peaks at 87.6 eV and 84.0 eV, corresponding to the spin–orbit split Au 4f 5/2 and Au 4f 7/2 components, respectively. These binding energies are consistent with metallic gold, confirming that gold is present entirely in its elemental form. The absence of additional peaks or shifts indicates that there is no significant oxidation or chemical interaction affecting the Au atoms [57,58].

### 2.3. TGA/DSC Thermal Analysis

**Figure 6:**
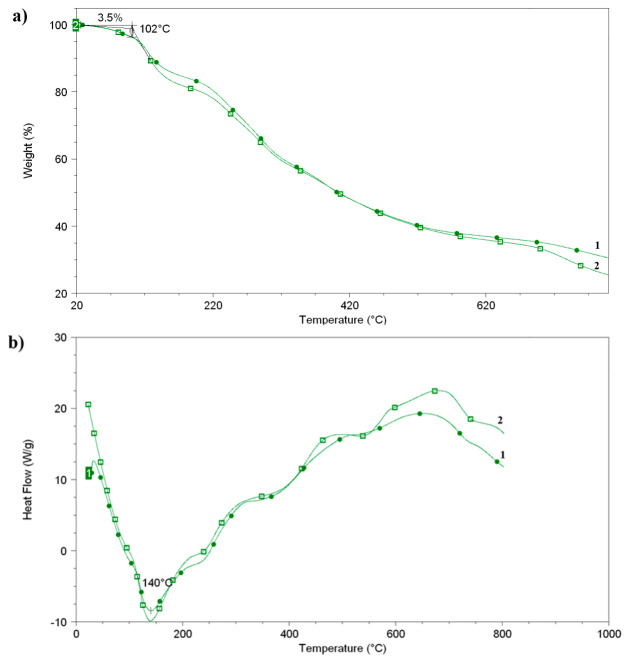
TGA thermal curves of samples **1** and **2** in temperature range 0–800 °C: (**a**) decomposition and (**b**) melting point.

The TGA curve of samples **1** and **2** (Figure 6a) demonstrates a total weight loss of about 3.5% at 102 °C, being solely due to the evaporation of physically adsorbed water. The TGA curves of samples **1** and **2** show no complete degradation at 820 °C. These results suggest that the residue (about 30%) in samples **1** and **2** most likely originates from carbon. Sample 1 has higher thermal stability than sample 2. The broad endothermic peaks from 120 °C to 170 °C (Figure 6b) could indicate a phase transformation caused by melting. The thermal degradation of samples **1** and **2** is characterized by a multiple-step process entailed by chain scission and decomposition reactions. The sequence of broad exothermic peaks (from 300 to 820 °C) originating from the degradation and decomposition of fulvic acid, humic substances, and phenolic compounds in samples **1** and **2** (Figure 6b) is in agreement with the research of other authors [59,60].

**Figure 7:**
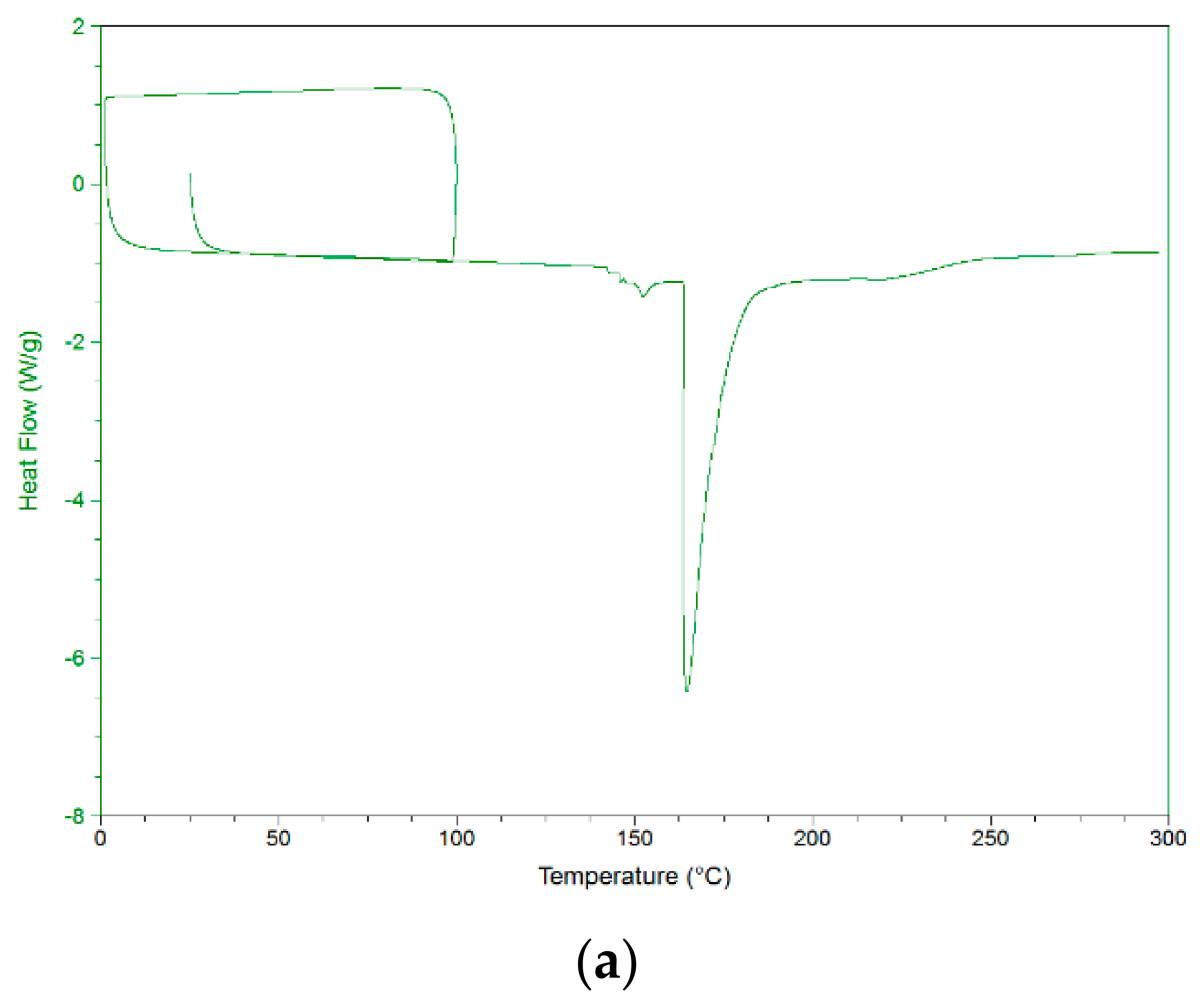

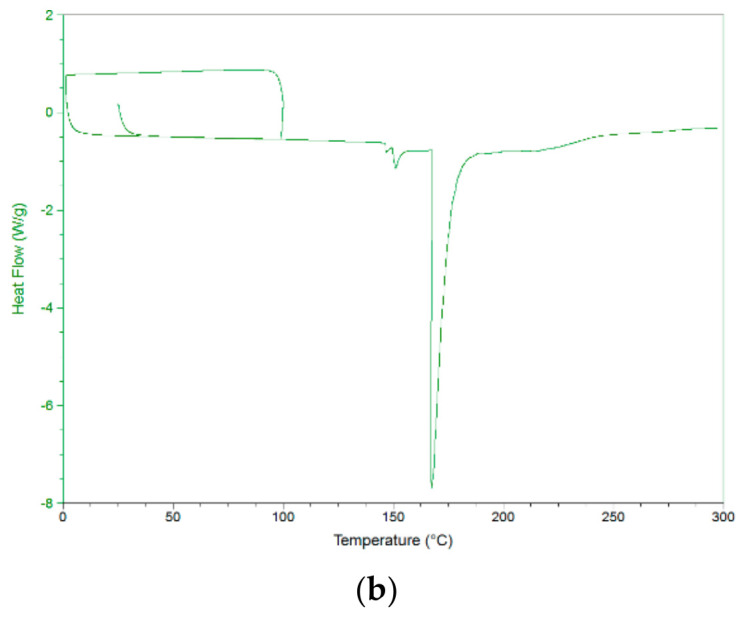
DSC curves in temperature range 0–300 °C: (**a**) sample **1**, and (**b**) sample **2**.

More precise thermal DSC analysis was performed in the temperature range from room temperature to 300 °C. The endothermic phase transition dominates the DSC thermogram of sample **1** (Figure 7a) with a sharp endothermic peak centered at 165.9 °C. Also, the endothermic phase transition dominates the DSC thermogram of sample **2** (Figure 7b) with a sharp endothermic peak centered at 169.1 °C. Endothermic peaks originate from the melting phase transformation of samples. The small difference in the melting temperature of samples **1** and **2** comes from the difference in composition and structure of the samples.

### 2.4. Antimicrobial Activity

The antimicrobial activity of the biosynthesized AuNPs and SeNPs was evaluated by determining their minimum inhibitory concentration (MIC) against a range of common microorganisms, which included pathogens responsible for human, animal, and plant diseases, mycotoxin producers, and food spoilage agents. As indicated in Table 2 and Table 3, all tested NPs inhibited the growth of the microorganisms, with MIC values ranging from 0.125 to 5 mg/mL. Regarding the tested Gram-positive and Gram-negative bacteria, the MIC for our compounds ranged from 0.125 to 5 mg/mL. The stronger antibacterial activity was found in SeNPs, which in low amounts inhibited all the species of bacteria. The lowest measured MIC value (0.125 mg/mL), and therefore the best antibacterial activity, was displayed for the SeNPs against *B*. *subtilis* and *S*. *aureus*. In contrast, *K*. *pneumoniae* showed the highest resistance to AuNPs (MIC = 5 mg/mL). The MIC related to the antifungal activity ranged from 0.125 to 5 mg/mL. Among the tested species, the most sensitive appeared to be *M*. *mucedo*, and then *P*. *italicum* and *G*. *candidum*. AuNPs showed consistently weaker antifungal effects, with MIC values mostly at 5 mg/mL. As a result of this study, SeNPs showed marked antimicrobial activity. Therefore, this biosynthesized compound can be a new, potential antimicrobial agent.

The antibacterial efficacy was assessed against common medications, specifically streptomycin (for bacteria) and ketoconazole (for fungi). The findings indicated that conventional antibiotics exhibited comparable efficacy compared to the tested samples, as illustrated in Table 2 and Table 3. DMSO exhibited no inhibitory effect on the examined organisms in the negative control.

In this study, it was observed that the tested biosynthesized NPs demonstrated moderate inhibitory effects on Gram-negative bacteria and fungi only at higher concentrations, whereas Gram-positive bacteria were more susceptible. This variation in sensitivity is expected due to the protective outer membrane characteristic of Gram-negative bacteria, which is absent in Gram-positive strains [61]. Fungi also exhibit higher resistance, largely due to the complex structure of their cell walls, which include glucans, chitin, and glycoproteins [62].

AuNPs and SeNPs are attractive because of their biocompatibility and distinctive characteristics, which are frequently augmented when made by green and biological approaches. Recent studies demonstrate that biogenic SeNPs can be obtained from several natural sources, including citrus fruits, resulting in potent antibacterial agents [63]. Contrary to literature reports that show relatively elevated MIC values for SeNPs against certain Gram-positive bacteria, including *Streptococcus pneumoniae* and MRSA (>50 ppm), the SeNPs evaluated in this study exhibited significantly enhanced antimicrobial efficacy, with MIC values as low as 0.125 mg/mL against *B. subtilis* and *S. aureus*. Likewise, whereas AuNPs are frequently noted to exhibit inconsistent antibacterial efficacy based on formulation, our findings demonstrated uniform activity, especially against *S. aureus*, *E. coli*, and *P. mirabilis* (MIC = 1.25 mg/mL). The enhanced antibacterial efficacy of SeNPs can be ascribed to their diminutive particle size and extensive surface area, which augment their contact with microbial membranes, resulting in the disruption of cellular activities and the production of reactive oxygen species (ROS) [64]. These results align with prior research demonstrating significant antibacterial properties of green-synthesized SeNPs against both Gram-positive and Gram-negative bacteria [65,66]. The green-synthesized SeNPs surpassed AuNPs in antibacterial and antifungal experiments, likely due to variations in physicochemical characteristics, redox potential, and interactions with microbial structures.

The present study successfully demonstrates a fully green, additive-free synthesis of AuNPs and SeNPs NPs using Himalayan shilajit as the sole reducing, stabilizing, and capping agent. The results highlight the critical role of urolithin A (**Uro-A**) content in shilajit for efficient nanoparticle formation and provide comprehensive physicochemical and biological characterization of the resulting nanomaterials. HPLC-MS analysis revealed that the methanol/ethyl acetate (MeOH/EA) solvent system was markedly superior for extracting **Uro-A** from shilajit, with sample **1** yielding 2.504 ppm compared to only 0.209 ppm in sample **2**. This difference directly correlated with nanoparticle synthesis efficiency: sample **1** produced high yields (90% for AuNPs and 96% for SeNPs), whereas sample **2** yielded only 15% and 34%, respectively. Control experiments using aqueous **Uro-A** solutions further confirmed its contribution to the reduction process. As a polar phenolic metabolite with strong antioxidant and metal-chelating properties, **Uro-A** (along with related humic/fulvic acids and other polyphenols in shilajit) likely acts as both a reducing agent for Au^3+^ and SeO_3_^2−^ ions and a capping/stabilizing ligand. The poor performance of non-polar (hexane) and polar (ethyl acetate) extracts underscores the importance of solvent polarity in liberating these bioactive constituents.

SEM analysis showed that both AuNPs and SeNPs predominantly exhibited spherical morphology with particle sizes predominantly in the 80–300 nm range and an average around 100 nm, consistent with the unimodal particle size distribution obtained by DLS. The relatively low negative zeta potential values (−2.2 mV for AuNPs and −4.8 mV for SeNPs) indicate moderate electrostatic stabilization, explaining the observed tendency toward agglomeration visible in high-magnification SEM images. Despite this, the particles remained sufficiently dispersed for practical applications. XRD and XPS analyses unequivocally confirmed the formation of elemental (zero-valent) Au and Se, with characteristic Bragg reflections and binding energies matching metallic gold and metalloid selenium, respectively. The low organic phase content (<5 wt% by XRD estimation) combined with FTIR evidence of surface-bound humic/fulvic acids, phenolic groups, and carbonyl moieties demonstrates successful biogenic capping by shilajit-derived biomolecules. The stronger IR absorption and higher carbon content observed in SeNPs suggest a thicker or more robust organic coating on selenium, likely due to its higher surface reactivity compared to gold. TGA/DSC data further supported the thermal stability of the shilajit matrix and the presence of organic components, with characteristic decomposition profiles of fulvic and humic substances.

The antimicrobial evaluation revealed that shilajit-capped SeNPs exhibited significantly superior activity compared to AuNPs against both bacterial and fungal strains (MIC values 0.125–1 mg/mL vs. 0.25–5 mg/mL). SeNPs were particularly effective against Gram-positive bacteria (*B. subtilis* and *S. aureus*, MIC 0.125 mg/mL) and certain fungi (*P. italicum* and *G. candidum*, MIC 0.125 mg/mL). This enhanced efficacy can be attributed to several synergistic factors: (i) the intrinsic antimicrobial properties of elemental selenium NPs, (ii) the smaller effective size and higher surface area facilitating membrane disruption and ROS generation, and (iii) the bioactive shilajit capping layer, which may contribute additional phenolic and humic-derived antimicrobial effects. The differential sensitivity between Gram-positive and Gram-negative bacteria aligns with the known protective role of the outer membrane in Gram-negatives. Although the observed MIC values are higher than those of conventional antibiotics (streptomycin and ketoconazole), the green-synthesized SeNPs represent a promising alternative or adjunctive agent, especially in the context of rising AMR. AuNPs generally have lower intrinsic antimicrobial activity than SeNPs [67]. The primary mechanism of antimicrobial action of SeNPs is ROS that damage the microbial cell wall [68]. AuNPs interfere with essential thiol enzymes in bacteria, which is their primary mechanism of antibacterial action [69].

These findings are consistent with recent literature on green-synthesized metallic/metalloid NPs, where plant- or biomolecule-derived capping agents enhance both stability and biological activity. However, the use of shilajit as a single, complex natural matrix offers advantages in simplicity, cost-effectiveness, and potential multi-component bioactivity. The mild reaction conditions (50 °C, aqueous medium) further emphasize the sustainability of the approach. In conclusion, the strong correlation between **Uro-A** content and synthetic efficiency, combined with favorable physicochemical properties and promising antimicrobial performance of SeNPs, positions shilajit-mediated synthesis as an attractive green nanotechnology platform. Future studies should focus on optimizing particle size and zeta potential (e.g., through pH or concentration adjustments), evaluating in vivo efficacy and toxicity, and exploring synergistic formulations for targeted biomedical applications.

### 2.5. Sustainability Aspects

Conventional synthesis generates toxic waste, volatile organic compounds, and heavy metal effluents, conflicting with circular economy principles. This method is additive-free, uses a renewable natural resource (shilajit from geological plant decomposition), operates in water at low temperature, and produces minimal hazardous waste. Shilajit purification involves simple dissolution, filtration, and concentration—low-impact steps. The biogenic capping not only stabilizes NPs but imparts additional bioactivity, potentially extending product lifespan and reducing dosage needs in applications. Low organic content (<5 wt% by XRD) in the final NPs, combined with natural degradability of the capping layer, supports better environmental fate compared to synthetic stabilizers. XPS and EDS confirm pure elemental Au/Se with natural organic coatings, minimizing secondary pollution risks. The Himalayan shilajit (asphaltum)-driven synthesis of AuNP and SeNP NPs presented in this study represents a fully green, additive-free, one-pot process that addresses key limitations of conventional physical and chemical nanoparticle fabrication methods. Traditional approaches often rely on hazardous reducing agents (e.g., sodium borohydride, hydrazine), organic solvents, high temperatures, and multi-step protocols, resulting in toxic byproducts, high energy consumption, and poor biocompatibility. In contrast, this method uses shilajit resin—a complex natural matrix rich in urolithins, fulvic/humic acids, and polyphenols—as the sole reducing, stabilizing, and capping agent under mild aqueous conditions (50 °C, 3 h). This delivers high yields, effective antimicrobial performance, and clear sustainability gains. Shilajit, sourced sustainably from Himalayan regions and available as biomaterial resin, serves as both precursor and stabilizer, eliminating expenses for synthetic chemicals, surfactants, or specialized equipment. The reaction uses inexpensive precursors (KAuCl_4_ or Na_2_SeO_3_), ultrapure water, and mild heating (50 °C), contrasting with energy-intensive physical methods (laser ablation, plasma) or chemical reductions requiring controlled atmospheres and high temperatures.

## 3. Discussion

This study presents a fully green and additive-free method for synthesizing gold and selenium NPs mediated by Himalayan shilajit as the sole reducing, stabilizing, and capping agent. By systematically comparing solvent extraction systems, it is demonstrated that a methanol/ethyl acetate mixture is significantly better for extracting urolithin A (**Uro-A**), a key bioactive phenolic metabolite, from shilajit. The developed one-pot synthesis at mild temperature (50 °C) successfully produced spherical AuNPs (80–250 nm) and SeNPs (80–300 nm) embedded within a continuous organic polymer matrix derived from shilajit. Comprehensive physicochemical characterization (SEM-EDS, DLS, zeta potential, FT-IR, XRD, TGA/DSC) revealed that both nanoparticle types are effectively capped by shilajit-derived biomolecules, with SeNPs exhibiting a distinctly thicker and more robust organic coating. This is evidenced by stronger IR absorptions (especially in the 1650–1600 cm^−1^ region), higher carbon content in EDS, and a more negative zeta potential (−4.8 mV vs. −2.2 mV for AuNPs), indicating stronger electrostatic and steric stabilization. High-resolution XPS spectra to determine the chemical states and electronic environments of selenium and gold in the sample confirmed their elemental form. Antimicrobial evaluation against a broad panel of pathogenic bacteria and fungi clearly established the better antimicrobial performance of shilajit-capped SeNPs. Minimum inhibitory concentrations reached as low as 0.125 mg/mL against *B. subtilis*, *S. aureus*, *P. italicum*, and *G. candidum*, outperforming AuNPs by up to 40-fold in certain strains. In particular, the shilajit-capped SeNPs emerge as promising candidates for the development of next-generation antimicrobial agents capable of addressing the escalating global threat of AMR.

## 4. Materials and Methods

### 4.1. Chemicals and Reagents

All chemicals and reagents were of analytical grade and used without further purification. Ultrapure water with a resistivity of 18.2 MΩ·cm at 25 °C was obtained from a Milli-Q water purification system (Millipore, Bedford, MA, USA) and used for the preparation of all aqueous solutions. Sodium chloride (NaCl, ≥99.5%, Sigma-Aldrich, St. Louis, MO, USA), potassium phosphate monobasic (KHPO_4_, ≥99%, Merck, Darmstadt, Germany), and potassium phosphate dibasic (K_2_HPO_4_, ≥98%, Fluka, Buchs, Switzerland) were used to prepare phosphate-buffered saline (PBS). For nanoparticle synthesis, potassium gold(III) chloride (KAuCl_4_, 98%) and sodium selenite (Na_2_SeO_3_, 99%) provided by Sigma were used. Tris(hydroxymethyl)aminomethane (Tris base, ≥99.9%, Sigma, St. Louis, MO, USA) was dissolved in ultrapure water and adjusted to the desired pH using hydrochloric acid (HCl, 37% *w*/*w*, VWR Chemicals, Radnor, PA, USA) or sodium hydroxide pellets (NaOH, ≥98%, Carl Roth, Karlsruhe, Germany). Organic solvents included absolute ethanol (≥99.8%, VWR Chemicals), HPLC-grade acetonitrile (≥99.9%, Fisher Scientific, Waltham, MA, USA), and HPLC-grade methanol (≥99.9%, Honeywell, Charlotte, NC, USA). Dimethyl sulfoxide (DMSO, ≥99.9%, Sigma-Aldrich) was used as a co-solvent. For chromatographic and mass spectrometric analyses, trifluoroacetic acid (TFA, ≥99%, Sigma, St. Louis, MO, USA) and formic acid (≥98%, Fluka, Buchs, Switzerland) were used as mobile phase modifiers. Samples of shilajit **1** and **2** originated from high-altitude (around 2000 m, Arunachal Pradesh) and low-altitude Himalayan areas (around 600 m, Himachal Pradesh), respectively. Raw samples were purified by a combination of dissolution, filtration, and concentration steps to remove mineral and organic impurities. The collected raw material was first manually cleaned to remove stones, soil, and plant debris, then crushed and dissolved in warm ultrapure water (200 mL per 50 g of raw material) to extract the soluble compounds. The mixture was continuously stirred at 60 °C for 24 h. The mixture was cooled to room temperature, then filtered; ethanol (100 mL, 96% *v*/*v*) was added, and stirring continued for another 10 h. The resulting dark brown suspension was allowed to stand so that insoluble particles sedimented, after which the solution was decanted and centrifuged for 10 min at 6000 rpm. The supernatant was sequentially filtered through a cascade of filters with pore sizes of 300, 150 and 50 μm. The filtrate was then decanted and concentrated by gentle heating (60 °C) under vacuum. The final samples were obtained as a dark brown, sticky resin.

### 4.2. Synthesis of AuNPs and SeNPs

For the synthesis, 0.5 g of shilajit sample 1 was dissolved in 20 mL of distilled water, and the solution was heated to 50 °C. Subsequently, 0.5 g of KAuCl_4_ or Na_2_SeO_3_ was added in one portion to the solution to produce AuNPs or SeNPs, respectively. After 3 h, the reaction mixture was cooled to room temperature and centrifuged at 6000 rpm for 10 min. The supernatant was carefully discarded, and the resulting NPs were washed three times with ultrapure water (redispersing the pellet by brief sonication (10 min at 50 °C each time)) to remove impurities. After the final wash with absolute ethanol, the purified NPs were redispersed in ultrapure water for further characterization and use. For analysis, the NPs were dried in a vacuum oven at 40 °C for 12 h. Control experiments were conducted with shilajit sample **2**, then with 5, 10, and 15% (*w*/*w*) of **Uro-A** (aq.) solution (20 mL) under identical reaction conditions. The mixture was centrifuged at 10,000 rpm to precipitate the particles from the solution. When shilajit **2** with low **Uro-A** content was employed, the isolated yields dropped to 15% (AuNPs) and 34% (SeNPs). On the other hand, using the **Uro-A** solution (15%, 20 mL) led to substantially improved yields of 50% and 58% for AuNPs and SeNPs, respectively.

### 4.3. Differential Scanning Calorimetry (DSC) Analysis

DSC analysis for samples was performed using DSC Q20 equipment (TA Instruments, New Castle, DE, USA) to investigate the thermal properties of the prepared samples (**1** and **2**). Indium reference samples and sapphire crystal provided by TA Instruments were used for cell constant and Cp calibration. Calorimetric experiments were carried out under a nitrogen atmosphere at a flow rate of 50 mL·min^–1^ with samples hermetically closed in aluminum pans. All samples were heated at a constant heating/cooling rate of 10 °C·min^–1^ from 25 to 100 °C, then cooled to –90 °C and reheated to 280 °C. Melting (Tm) and crystallization (Tc) points were determined as the onset temperatures of the phase changes, and the corresponding enthalpies of the phase transitions were noted.

### 4.4. Thermogravimetric Analysis (TGA)

TGA was performed using a SDT Q600 simultaneous TG/DSC analyzer (TA Instruments, New Castle, DE, USA). Approximately 10 mg of each sample was placed in an open alumina pan and heated from 30 °C to 800 °C at a constant rate of 10 °C min^−1^ under a nitrogen flow of 50 mL min^−1^.

### 4.5. Scanning Electron Microscopy (SEM) Measurement

Specimens were examined by a JSM-6460LV scanning electron microscope operating (JEOL Ltd., Tokyo, Japan) at 25 kV. Local chemical analyses were performed on SEM equipped with an energy dispersive X-ray analysis (EDS) device, using an INCA Microanalysis system (Oxford Instruments, Abingdon, Oxfordshire, UK). The specimens were previously coated with gold using the Bal-tec SCD-005 device.

### 4.6. Dynamic Light Scattering (DLS) Measurements

DLS measurements [particle size distribution (PSD), and zeta potential] were performed on an Litesizer DLS 701 (Anton Paar GmbH, Graz, Austria) with Multi-Angle Particle Sizing (MAPS) capability. Approximately 10.0 mL of sample was sonicated and then transferred into a disposable omega cuvette for PSD measurements or a folded capillary zeta cell for ζ-potential. Samples were thermally equilibrated at 25.0 ± 0.1 °C for 1 min. Data were analyzed using Kalliope™ professional software.

### 4.7. X-Ray Diffraction (XRD) Analysis

Powder XRD patterns were recorded on a MiniFlex 300/600 diffractometer (Rigaku Corporation, Tokyo, Japan) using Cu Kα radiation (λ = 1.5406 Å) generated at 40 kV and 15 mA. The measurement was performed in Bragg–Brentano (θ/2θ) geometry with a standard sample holder. A bent graphite monochromator was employed on the diffracted beam side, while no monochromator was used on the incident beam. The optical configuration included a 10.0 mm incident beam height slit, a 0.625° divergence slit, a 1.250° scatter slit, and a 0.3 mm receiving slit. Data were collected in continuous scan mode from 30° to 100° (2θ) with a step size of 0.020° and a scan speed of 5.0° min^−1^. The detector used was a Rigaku SC-70 scintillation counter.

### 4.8. X-Ray Photoelectron Spectroscopy (XPS) Analysis

The samples were examined using a XP50M X-ray source and a Focus 500 monochromator, in combination with a PHOIBOS 100/150 hemispherical analyzer (SPECS Surface Nano Analysis GmbH, Berlin, Germany). Measurements were carried out with Al Kα radiation (1486.74 eV), with the source operating at 12.5 kV and 32 mA. High-resolution spectra for the Se 3d and Au 4f were collected using a constant pass energy of 20 eV, a step size of 0.1 eV, and a dwell time of 2 s, with acquisition performed in FAT mode. During data collection, the base pressure was kept at 4 × 10^−8^ mbar. Binding energy calibration was achieved by setting the C 1 s peak to 284.8 eV.

### 4.9. LC-MS/MS Analysis

Analytical standards of **Uro-A** and **Uro-B** were obtained from Toronto Research Chemicals (Toronto, ON, Canada). HPLC-grade methanol and water were purchased from Sigma-Aldrich (St. Louis, MO, USA). Formic acid (p.a.) was obtained from Merck (Darmstadt, Germany). All other chemicals were purchased by Sigma. The LC-MS/MS system consisted of Shimadzu (Kyoto, Japan) components: two LC-40D xs UPLC pumps connected in binary gradient mode, a DGU-405 degassing unit, a SIL-40C xs autosampler, a CTO-40C column oven, an SCL-40 system controller, and an LC-MS 8045 triple-quadrupole mass spectrometer. Phenomenex Kinetex C18 reversed-phase column (50 × 2.1 mm, 2.6 µm) was used for chromatographic separation. Each sample (1 g) was extracted using freshly distilled hexane, ethyl acetate (EA), or a methanol/ethyl acetate (MeOH/EA) mixture (20 mL, 1:1, *v*/*v*). The resulting extracts were centrifuged at 6000 rpm, filtered through 0.45 µm nylon syringe filters to remove particulate matter, and transferred to HPLC vials for analysis. For optimal sensitivity, 10 nanograms of each sample were injected, with autotune software fine-tuning the instrument parameters to maximize detection accuracy. Limit of detection for **Uro-A** and **Uro-B** were 50 and 35 ppb in MeOH/EA extract, respectively. Calibration curves (Figure S1) were established for the standards **Uro-A** and **Uro-B** by injecting solutions prepared in a MeOH/EA (1:1, *v*/*v*) mixture, corresponding to mass concentrations of 5, 10, 15, and 20 mg/kg. The total run time for each analysis was maintained at 10 min, ensuring efficient and precise quantification of the target compounds. The mass spectrometer operated in MRM mode, and the following transitions were determined: **Uro-A**: 227 > 198 CE 35 V; 227 > 154.1 CE 30 V and **Uro-B**: 211 > 139 CE 32 V; 211 > 167 CE 33 V (Figures S2–S7).

### 4.10. Fourier-Transform Infrared Spectroscopy (FT-IR)

ATR FT-IR was performed on a Nicolet iS10 FT-IR Spectrometer (Thermo Scientific Instruments, Waltham, MA, USA) equipped with a DTGS detector. Spectra were collected in the range of 4000–400 cm^−1^ at a resolution of 4 cm^−1^ using Norton-Beer (medium) apodization. Samples were first dried under vacuum and then directly placed onto the diamond ATR crystal.

### 4.11. Antimicrobial Analysis

The antimicrobial potential of the investigated compounds was evaluated against a panel of bacterial and fungal strains obtained from the American Type Culture Collection (ATCC). The bacterial species included Bacillus subtilis (ATCC 6633), *Staphylococcus aureus* (ATCC 25923), *Escherichia coli* (ATCC 25922), *Proteus mirabilis* (ATCC 29906), and *Klebsiella pneumoniae* (ATCC 70063). The antifungal activity was assessed using *Aspergillus niger* (ATCC 16888), *Penicillium italicum* (ATCC 10454), *Mucor mucedo* (ATCC 20094), *Candida albicans* (ATCC 10259), and *Geotrichum candidum* (ATCC 34614). Bacterial strains were cultivated on Mueller–Hinton agar, whereas fungal strains were maintained on Sabouraud dextrose agar (Torlak, Belgrade, Serbia). Bacterial inocula were prepared from 24-h-old cultures incubated at 37 °C and adjusted to a concentration of approximately 10^8^ CFU/mL, corresponding to the 0.5 McFarland standard. Fungal suspensions were obtained from spores harvested from 3–7-day-old cultures grown on Sabouraud dextrose agar and suspended in sterile distilled water to achieve a final concentration of about 10^6^ CFU/mL, in accordance with NCCLS guidelines [70].

The minimum inhibitory concentrations (MICs) were determined using the microdilution method in sterile 96-well microplates [71]. Resazurin was employed as a viability indicator for bacterial growth, while fungal inhibition was assessed by visual inspection. Stock solutions of the tested compounds were prepared in 10% DMSO, followed by serial twofold dilutions to obtain concentrations ranging from 50 to 0.195 mg/mL in Müller–Hinton broth for bacteria and Sabouraud dextrose broth for fungi. Afterward, standardized microbial suspensions were added to each well, together with resazurin in the case of bacterial assays. The microplates were incubated at 37 °C for 24 h for bacterial strains and at 28 °C for 72 h for fungal strains. For bacteria, the MIC was defined as the lowest concentration at which no color change of resazurin from blue to pink was observed. In fungal assays, the MIC corresponded to the lowest concentration that visibly suppressed mycelial growth. Streptomycin and ketoconazole were used as reference antibiotics for bacteria and fungi, respectively, while DMSO served as the negative control to verify the absence of solvent effects.

### 4.12. Statistical Analysis

All data was presented as the mean of the results of three parallel measurements. Prior to statistical analysis, the normality of data distribution was assessed using the Shapiro–Wilk test. Since the data showed a normal distribution (*p* > 0.05), statistical analyses were performed using IBM SPSS Statistics version 26 (IBM Corp., Armonk, NY, USA). Student’s *t*-test was used to determine the statistical significance of differences between the control and the samples, as well as among the samples. Differences were considered statistically significant at *p* < 0.05.

## Figures and Tables

**Table 1:** Quantitative data for **Uro-A**. Concentration is expressed as mean ± standard deviation (SD) from triplicates. **Uro-B** is not detected.

		Uro-A (ppm)	
	Hexane	EA	MeOH/EA
Sample			
**1**	0.002 ± 0.001	0.010 ± 0.004	2.504 ± 0.147
**2**	0.005 ± 0.001	0.003 ± 0.001	0.209 ± 0.026

**Table 2:** The antibacterial activity of the tested compounds.

Tested Compounds	*Bacillus subtilis*	*Staphylococcus aureus*	*Escherichia coli*	*Proteus mirabilis*	*Klebsiella pneumoniae*
	MIC (mg/mL)				
AuNPs ^a^	2.5	1.25	1.25	1.25	5
SeNPs ^b^	0.125	0.125	0.5	0.25	1
Sample **1** ^a^	20	/	/	20	/
Sample **2** ^a^	/	/	/	20	/
Streptomycin	0.016	0.031	0.062	0.062	0.016

In all cases the SD is ± 0.001; ^a^—Values in the same column with different letters are significantly different (*p* < 0.05); ^b^—There is a statistically significant difference between tested compounds and the control (*p* < 0.05).

**Table 3:** The antifungal activity of the tested compounds.

Tested Compounds	*Candida albicans*	*Aspergillus niger*	*Penicillium italicum*	*Mucor mucedo*	*Geotrichum candidum*
	MIC (mg/mL)				
AuNPs ^a^	5	5	5	2.5	5
SeNPs	1 ^a^	0.5 ^a^	0.125 ^b^	0.5 ^b^	0.125 ^b^
Sample **1** ^a^	20	20	20	20	20
Sample **2** ^a^	20	20	20	20	20
Ketoconazole	0.039	0.078	0.156	0.156	0.078

In all cases the SD is ± 0.001; ^a^—Values in the same column with different letters are significantly different (*p* < 0.05); ^b^—There is a statistically significant difference between tested compounds and the control (*p* < 0.05).

## Data Availability

The original contributions presented in this study are included in the article. Further inquiries can be directed to the corresponding author.

## Supplementary Materials

The following supporting information can be downloaded at https://www.mdpi.com/article/10.3390/molecules31152649/s1, Figure S1: Chromatograms of standards **Uro-A** and **Uro-B** and calibration curve. Figure S2: Sample **1** hexane extract. Figure S3: Sample **2** hexane extract. Figure S4: Sample **1** EA extract. Figure S5: Sample **2** EA extract. Figure S6: Sample **1** EA/MeOH extract. Figure S7: Sample **2** EA/MeOH extract. Figure S8: Particle size distribution of AuNPs (left) and SeNPs (right), presence of fractions in %.

## Abbreviations

The following abbreviations are used in this manuscript:

AuNPs	Gold NPs
SeNPs	Selenium NPs
Uro-A	Urolithin A
SEM	Scanning Electron Microscopy
EDS	Energy Dispersive X-ray Spectroscopy
PSD	Particle Size Distribution
ZP	Zeta Potential
XRD	X-ray Diffraction
FTIR	Fourier-Transform Infrared Spectroscopy
XPS	X-ray Photoelectron Spectroscopy
DSC	Differential Scanning Calorimetry
TGA	Thermogravimetric Analysis
MIC	Minimum Inhibitory Concentration
PBS	Phosphate-Buffered Saline
DLS	Dynamic Light Scattering
MeOH/EA	Methanol/Ethyl Acetate
EA	Ethyl Acetate
MRM	Multiple Reaction Monitoring
