# Supplementary material for: Himalayan Asphaltum-Driven Synthesis of Gold and Selenium Nanoparticles: Investigation of Antimicrobial Activity and Urolithin Levels
Source: Molecules. 2026 Jul 29;31(15):2649. doi: 10.3390/molecules31152649 (PMC13468206; doi:10.3390/molecules31152649)
Supplement: Supplementary file 1 [file molecules-31-02649-s001.zip › molecules-4370383-supplementary.pdf]

Electronic Supporting Information

associated with the paper

Himalayan Asphaltum-driven synthesis of Gold and Selenium Nanoparticles: Investigation of Antimicrobial Activity and Urolithin Levels

Nenad Janković,*[1] Nodari Rizun,[2] Srđan Stefanović,[3] Lazar Rakočević,[4] Jovana Matić,[1] Sebastian Baloš,[5] Marijana Kosanić,[6] Nenad Ignjatović[7]

[1]University of Kragujevac, Institute for Information Technologies Kragujevac, Department of Science, Liceja Kneževine Srbije 1A, 34000 Kragujevac, Serbia

[2]PÜRBLACK Inc.Spring, Texas, USA

[3]Institute of Meat Hygiene and Technology, Kaćanskog 13, 11000 Belgrade, Serbia

[4]Institute of nuclear sciences Vinča, University of Belgrade, Department of Atomic Physics, Mike Petrovića Alasa 12-14, 11000 Belgrade, Serbia

[5]Faculty of Technical Sciences, University of Novi Sad, Trg Dositeja Obradovića 6, Novi Sad 21000, Serbia

[6]University of Kragujevac, Faculty of Science, Department of Biology and Ecology, Radoja Domanovića 12, 34000 Kragujevac, Serbia

[7]Institute of Technical Sciences of the Serbian Academy of Science and Arts, Knez Mihailova 35/IV, P.O. Box 377, Belgrade, Serbia

MS Chromatogram
250411 Std urolithins 1

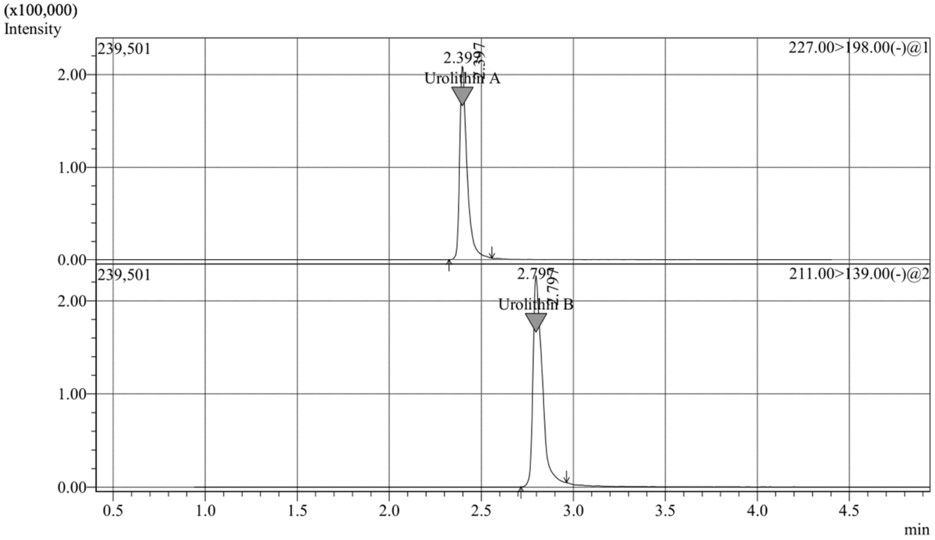

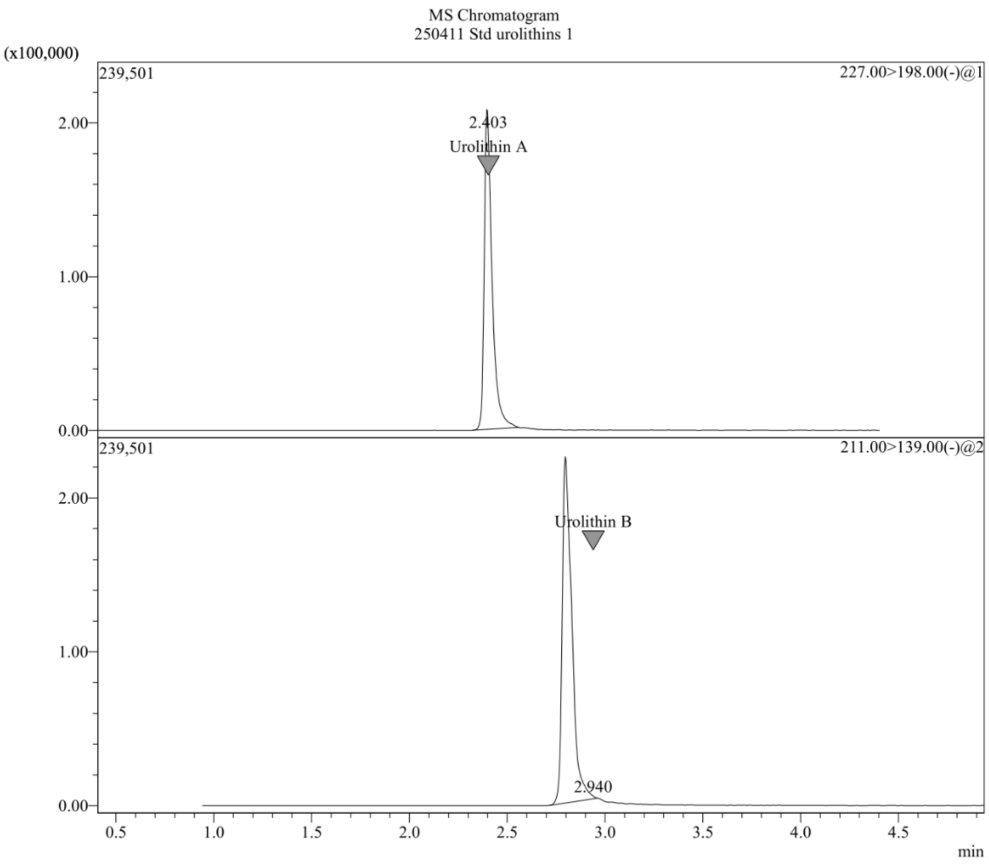

MS Chromatogram
250411 Std urolithins 1
(x100,000)
239,501
227.00>198.00(-)@1
2.403
Urolithin A
2.00
1.00
0.00
239,501
211.00>139.00(-)@2
Urolithin B
2.940
2.00
1.00
0.00
0.5
1.0
1.5
2.0
2.5
3.0
3.5
4.0
4.5
min

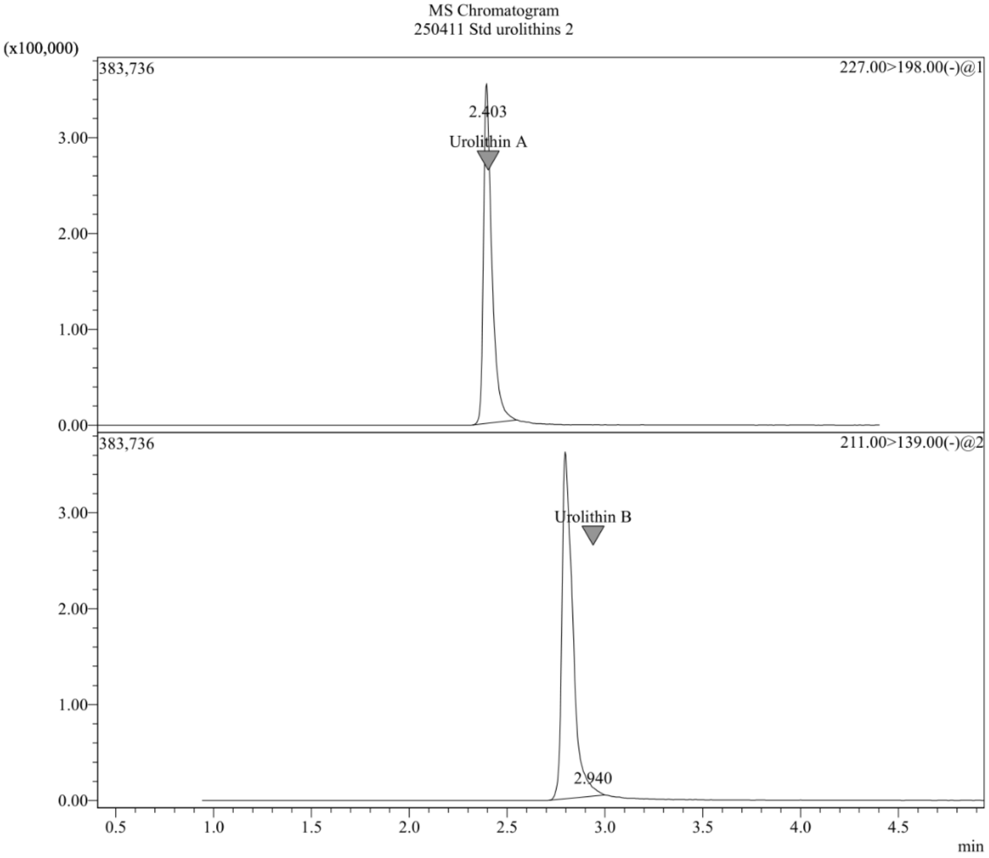

MS Chromatogram
250411 Std urolithins 2
(x100,000)
383,736
227.00>198.00(-)@1
2.403
Urolithin A
383,736
211.00>139.00(-)@2
Urolithin B
2.940
3.00
2.00
1.00
0.00
0.5
1.0
1.5
2.0
2.5
3.0
3.5
4.0
4.5
min

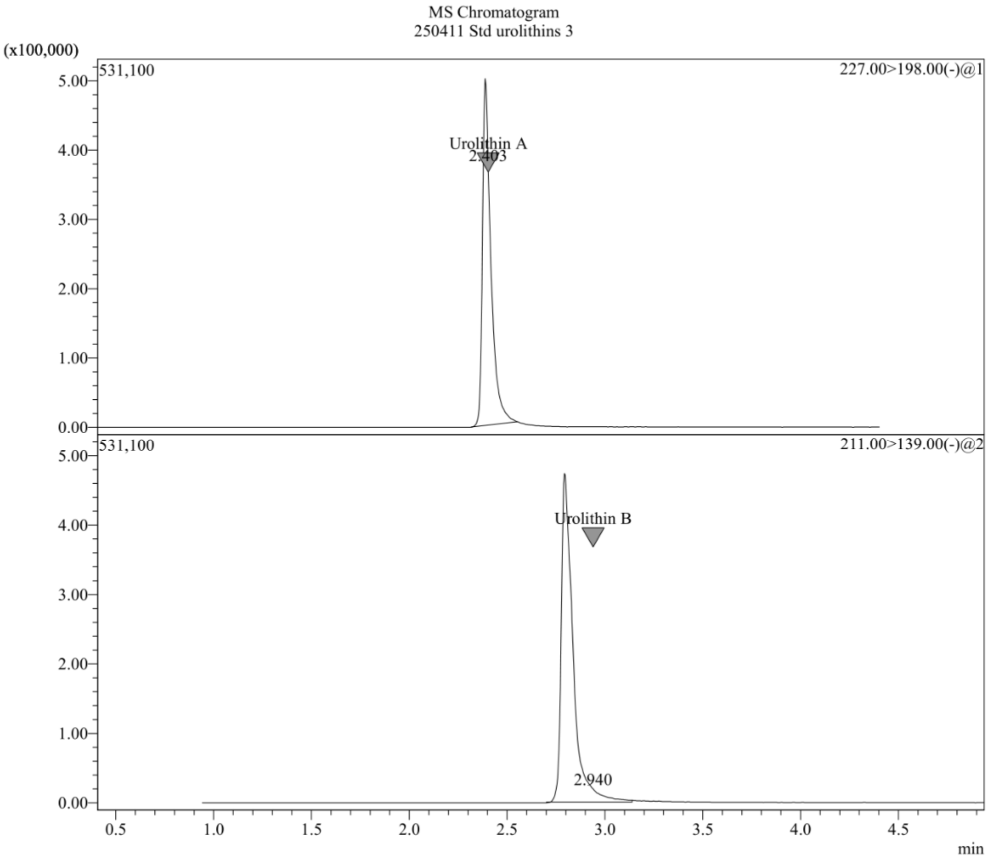
MS Chromatogram
250411 Std urolithins 3
(x100,000)
531,100
227.00>198.00(-)@1
Urolithin A
2.403
531,100
211.00>139.00(-)@2
Urolithin B
2.940
0.00
1.00
2.00
3.00
4.00
5.00
0.5
1.0
1.5
2.0
2.5
3.0
3.5
4.0
4.5
min

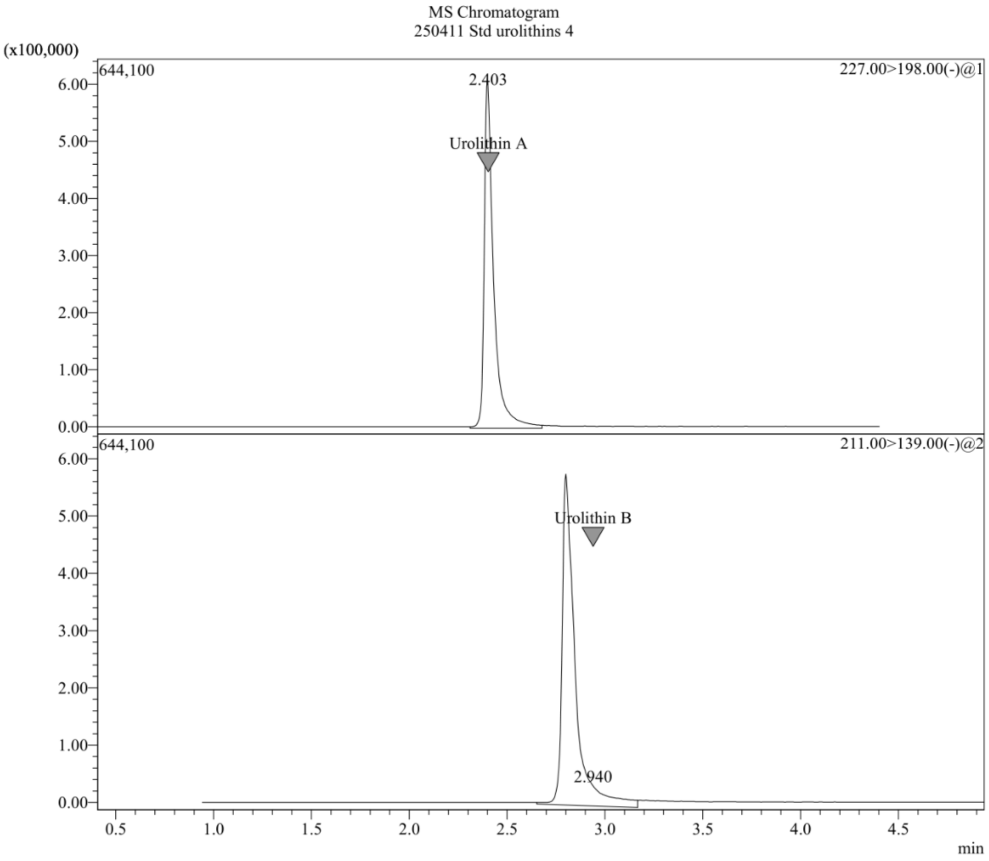

MS Chromatogram
250411 Std urolithins 4
(x100,000)
644,100
227.00>198.00(-)@1
2.403
Urolithin A
644,100
211.00>139.00(-)@2
Urolithin B
2.940
6.00
5.00
4.00
3.00
2.00
1.00
0.00
0.5
1.0
1.5
2.0
2.5
3.0
3.5
4.0
4.5
min

Calibration Curve

ID# : 1 m/z : 227.00>198.00
Name : Urolithin A
Quantitative Method : External Standard
Function : f(x)=152980*x+0
Rr1=0.9999620 Rr2=0.9999240
MeanRF: 1.553867e+005 RF SD: 3.934229e+003 RF %RSD: 2.531896
FitType : Linear
ZeroThrough : Through
Weighted Regression : None
Offset Correction : Off
Quantitative Method : External Standard

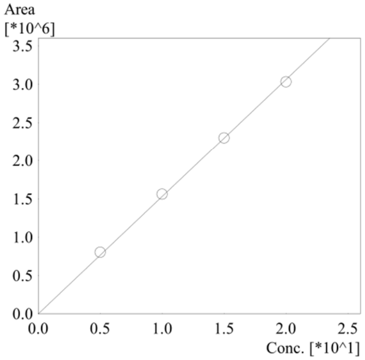

#	Conc.(Ratio)	MeanArea
1	5	802735
2	10	1561554
3	15	2299905
4	20	3030347

Area
802735
1561554
2299905
3030347

ID# : 2 m/z : 211.00>139.00
Name : Urolithin B
Quantitative Method : External Standard
Function : f(x)=173282*x+0
Rr1=0.9998028 Rr2=0.9996056
MeanRF: 1.774246e+005 RF SD: 7.735879e+003 RF %RSD: 4.360094
FitType : Linear
ZeroThrough : Through
Weighted Regression : None
Offset Correction : Off
Quantitative Method : External Standard

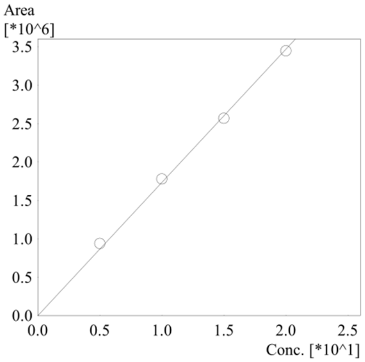

#	Conc.(Ratio)	MeanArea
1	5	940710
2	10	1779979
3	15	2568473
4	20	3446538

Area
940710
1779979
2568473
3446538

Figure S1. Chromatograms of standards **Uro-A** and **Uro-B** and calibration curve

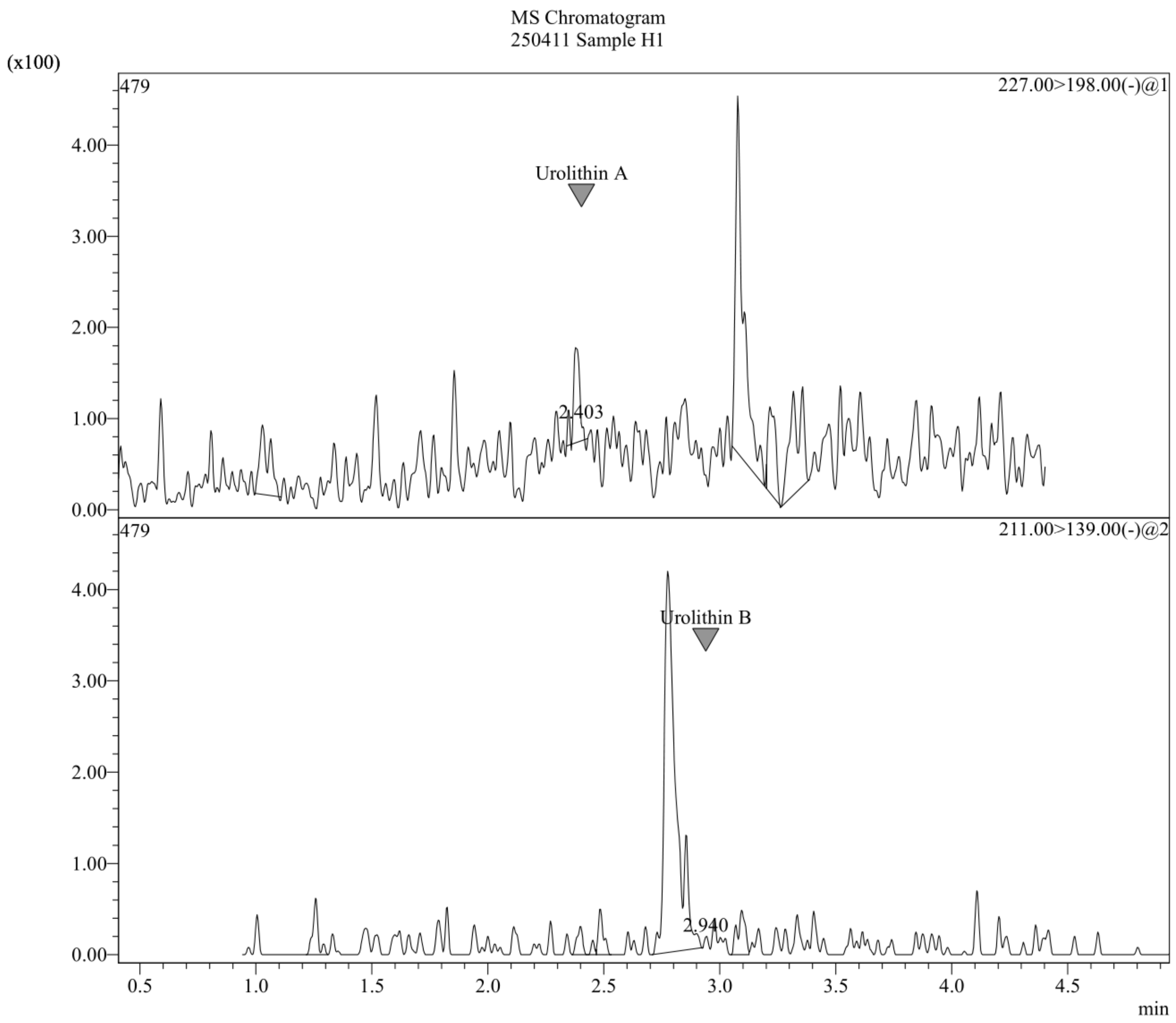

Figure S2. Sample **1** hexane extract

,

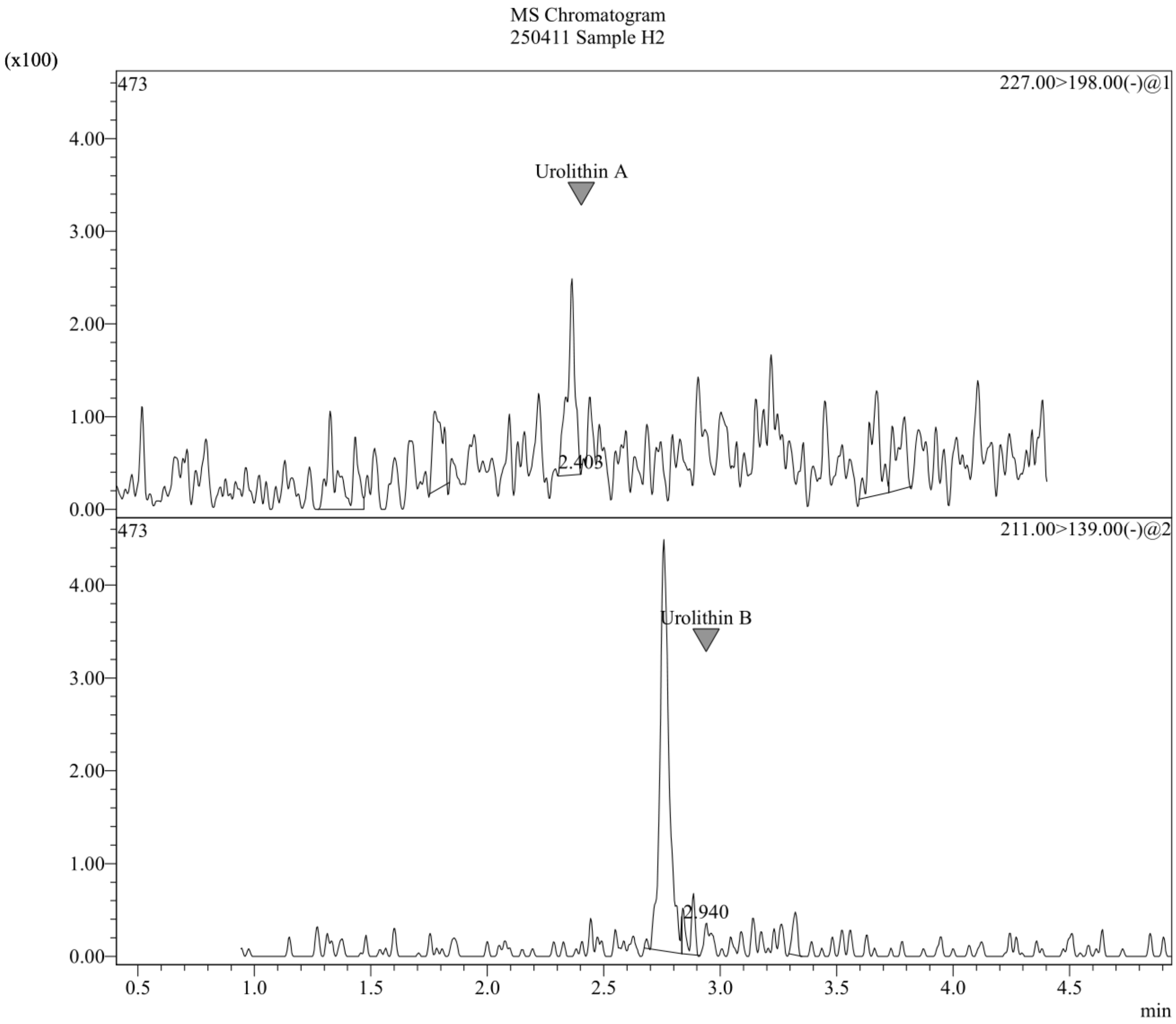

Figure S3. Sample **2** hexane extract

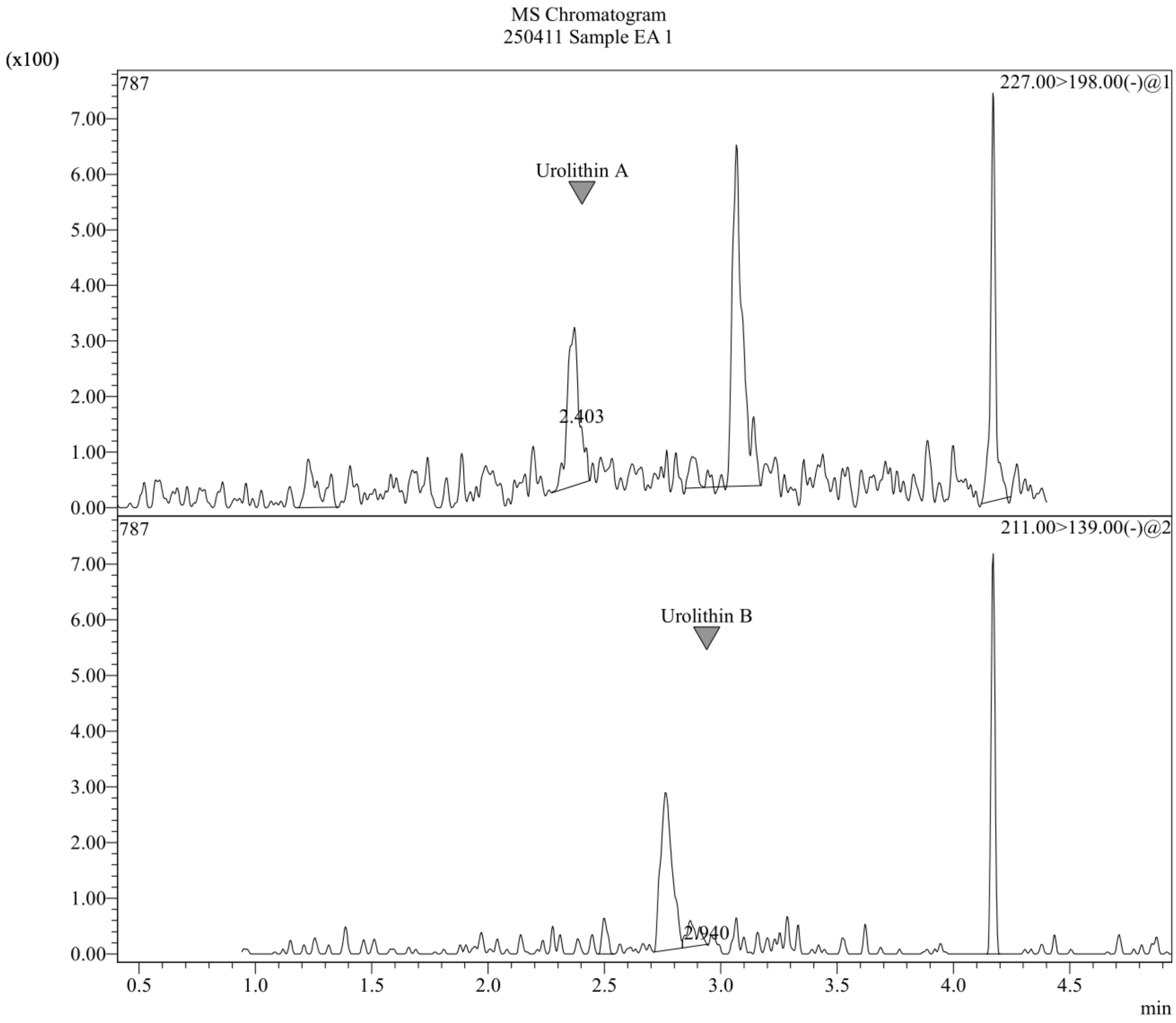

Figure S4. Sample **1** EA extract

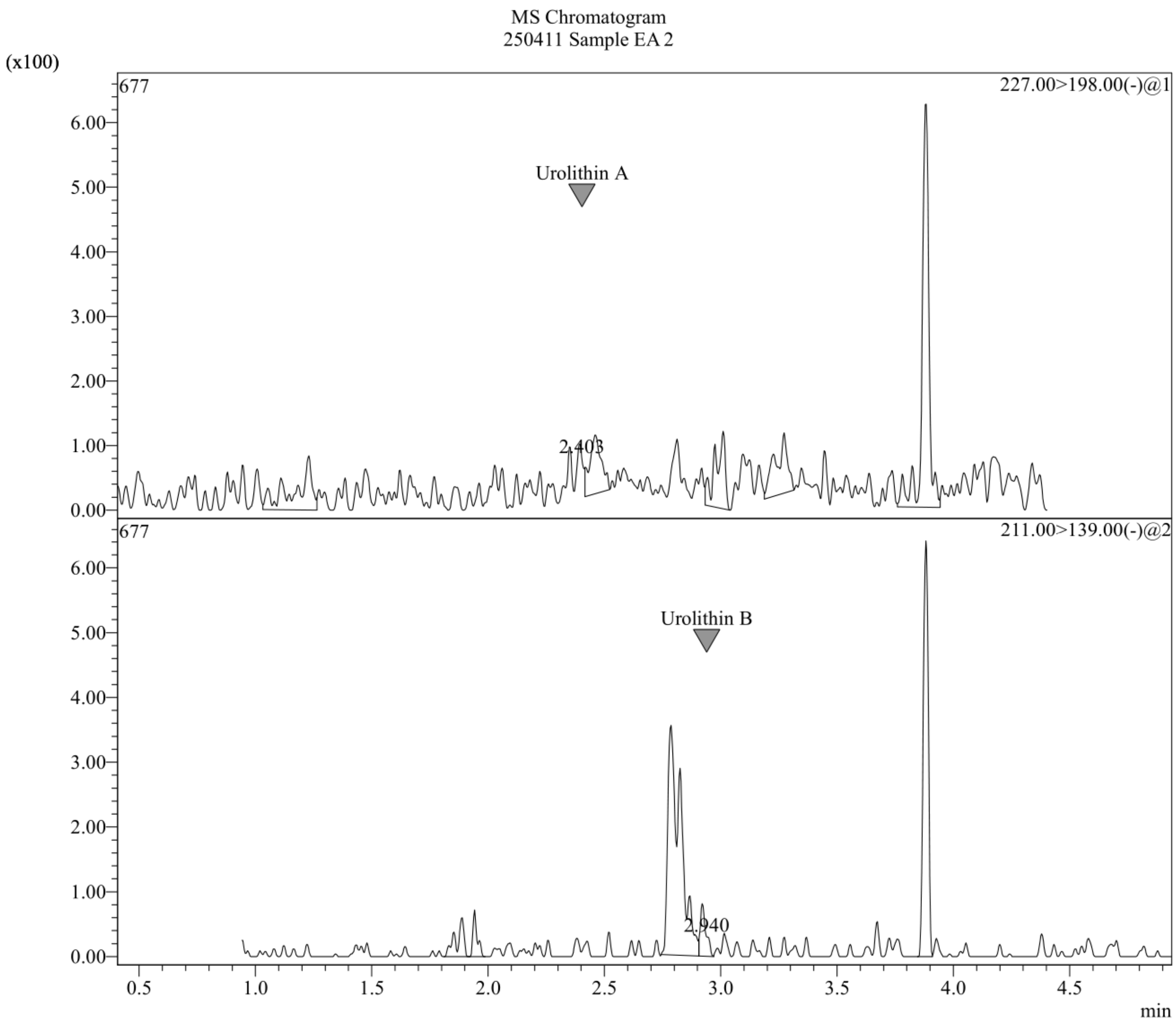

Figure S5. Sample **2** EA extract

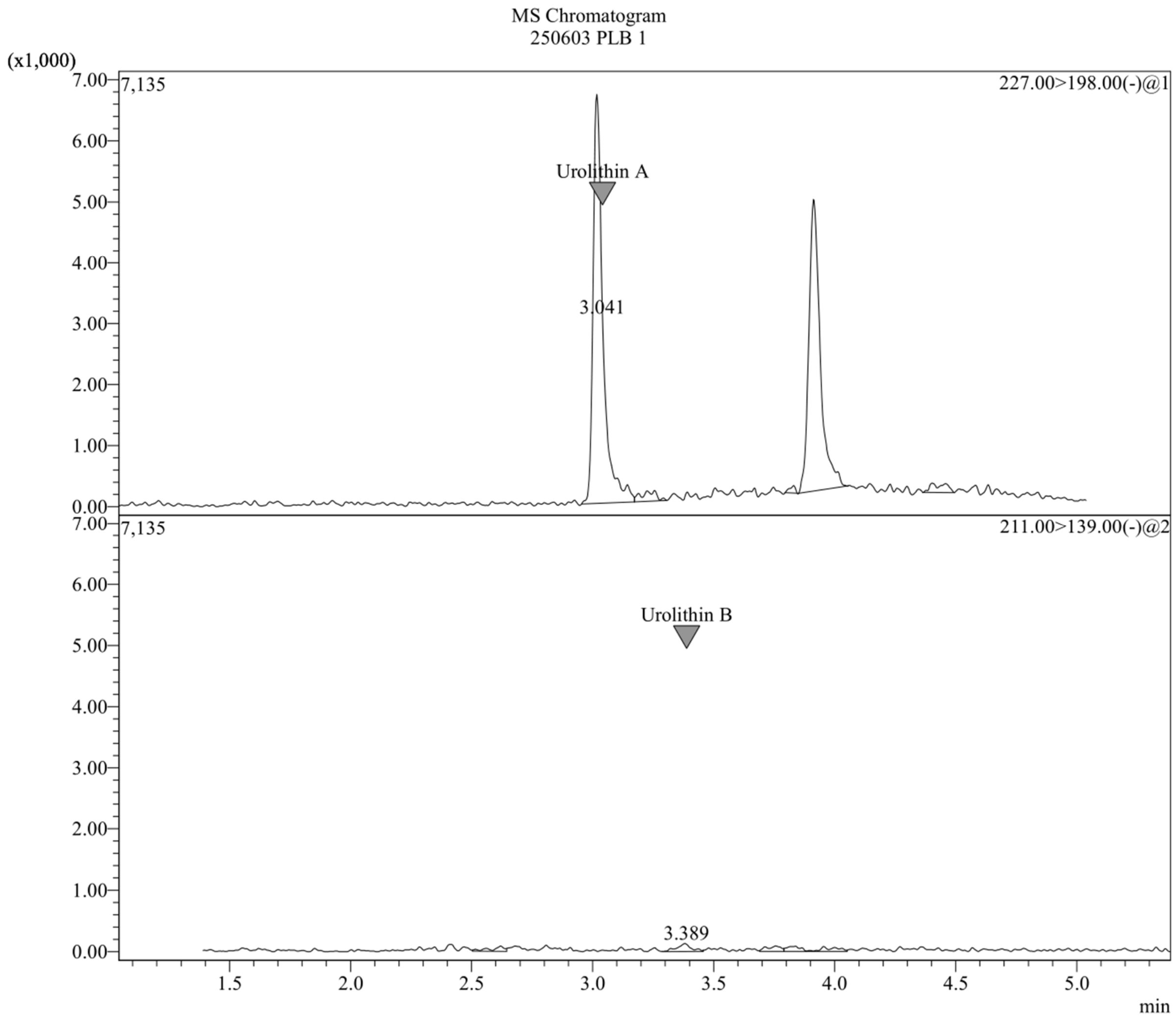

Figure S6. Sample **1** EA/MeOH extract

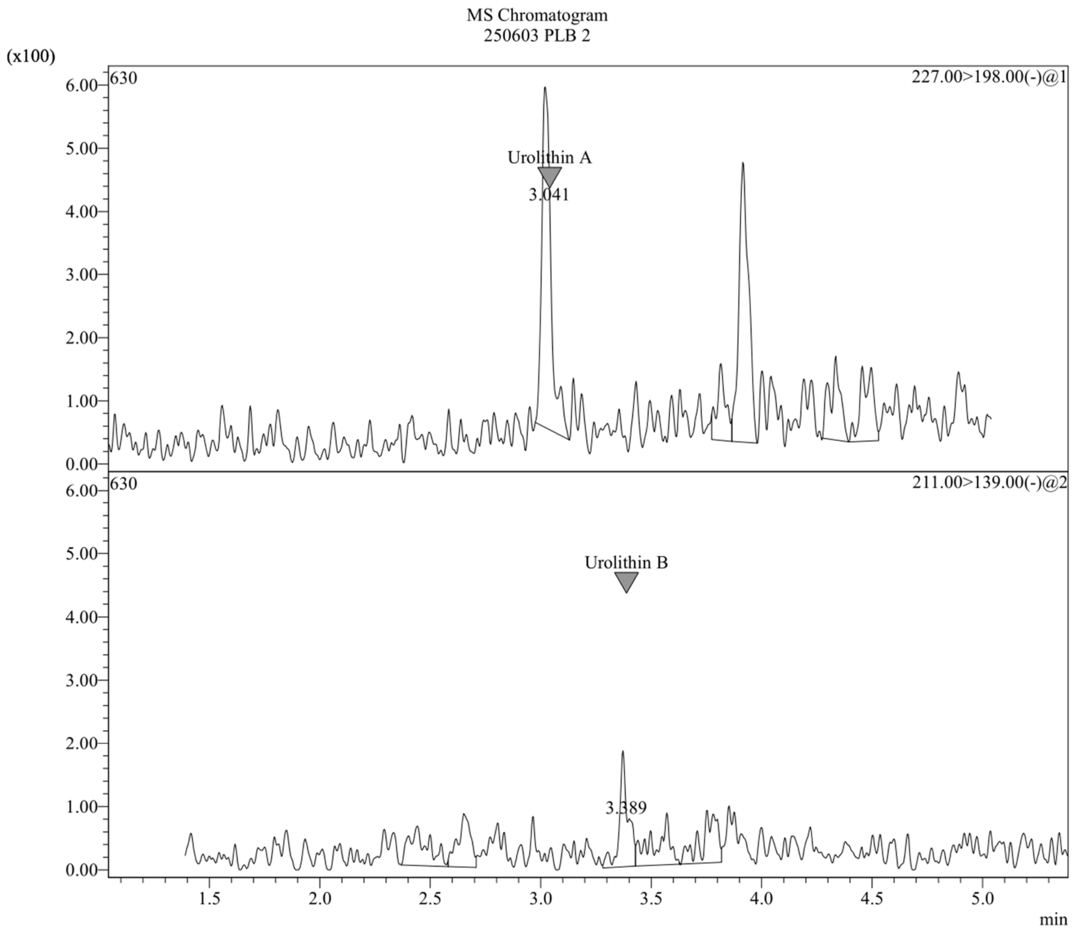

Figure S7. Sample **2** EA/MeOH extract

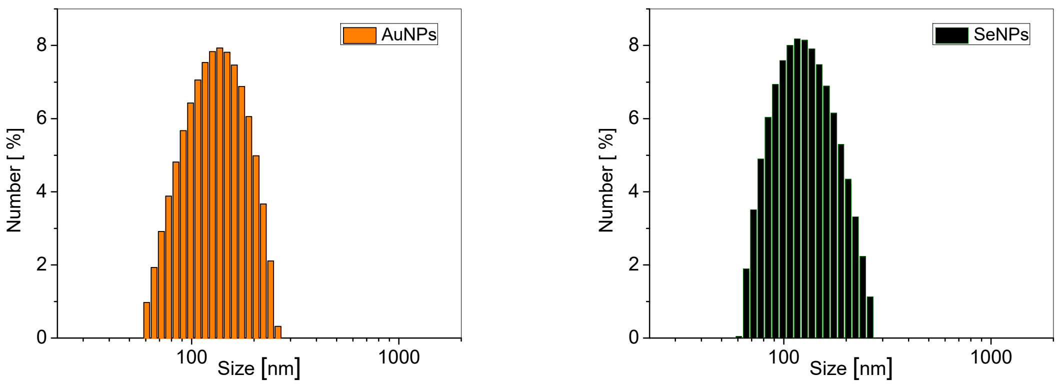

Figure S8. Particle size distribution of AuNPs (left) and SeNPs (right), presence of fractions in %
